# Auxin fluxes through plasmodesmata modify root-tip auxin distribution

**DOI:** 10.1242/dev.181669

**Published:** 2020-03-30

**Authors:** Nathan L. Mellor, Ute Voß, George Janes, Malcolm J. Bennett, Darren M. Wells, Leah R. Band

**Affiliations:** 1 Division of Plant and Crop Sciences, School of Biosciences, University of Nottingham, Sutton Bonington Campus, Loughborough LE12 5RD, UK; 2 Centre for Mathematical Medicine and Biology, School of Mathematical Sciences, University of Nottingham, Nottingham NG7 2RD, UK

**Keywords:** Auxin transport, Plasmodesmata, Mathematical modelling, Root biology

## Abstract

Auxin is a key signal regulating plant growth and development. It is well established that auxin dynamics depend on the spatial distribution of efflux and influx carriers on the cell membranes. In this study, we employ a systems approach to characterise an alternative symplastic pathway for auxin mobilisation via plasmodesmata, which function as intercellular pores linking the cytoplasm of adjacent cells. To investigate the role of plasmodesmata in auxin patterning, we developed a multicellular model of the *Arabidopsis* root tip. We tested the model predictions using the DII-VENUS auxin response reporter, comparing the predicted and observed DII-VENUS distributions using genetic and chemical perturbations designed to affect both carrier-mediated and plasmodesmatal auxin fluxes. The model revealed that carrier-mediated transport alone cannot explain the experimentally determined auxin distribution in the root tip. In contrast, a composite model that incorporates both carrier-mediated and plasmodesmatal auxin fluxes re-capitulates the root-tip auxin distribution. We found that auxin fluxes through plasmodesmata enable auxin reflux and increase total root-tip auxin. We conclude that auxin fluxes through plasmodesmata modify the auxin distribution created by efflux and influx carriers.

## Abstract (precis)

**Highlighted Article:** A multicellular model of auxin transport in the *Arabidopsis* root tip reveals the importance of intercellular plasmodesmata in maintaining auxin pattern and fluxes.

## INTRODUCTION

The plant hormone auxin regulates plant growth and plays a key role in many developmental responses (Benjamins and Scheres, 2008). Within the plant root, auxin controls root growth rate (Blilou et al., 2005; Rahman et al., 2007), lateral root development (Péret et al., 2009), root hair growth (Pitts et al., 1998; Knox et al., 2003; Jones et al., 2009), meristem length (Di Mambro et al., 2017), vasculature patterning (Bishopp et al., 2011; De Rybel et al., 2014), and adaptive responses such as gravitropism (Bennett et al., 1996) and halotropism (Galvan-Ampudia et al., 2013; Van den Berg et al., 2016). Precise knowledge of the auxin dynamics within the root tip is essential to understand how these processes are controlled.

Auxin is transported through plant tissues both by passive diffusion and by specialized proteins located on cell membranes (which are often referred to as ‘carriers’). The plasma membrane-localised PIN proteins facilitate efflux of anionic auxin from the cell cytoplasm to the apoplast and are typically polar localised within the root tip (Blilou et al., 2005). AUX1/LAX symporters facilitate the influx of anionic auxin (by co-transporting two protons with each anion of auxin; Lomax et al., 1985) and are typically non-polar within the root tip (Swarup et al., 2005). ABCB transporters primarily transport auxin out of the cell cytoplasm and appear to be non-polar (Geisler et al., 2005, 2017; Terasaka et al., 2005; Wu et al., 2007; Spalding, 2013). The cellular and subcellular distributions of these carriers have been shown to play a major role in controlling organ-scale auxin distributions and flux patterns (Swarup et al., 2005; Blilou et al., 2005; Lewis et al., 2007; Grieneisen et al., 2007; Band et al., 2014). Within the plant root, the carrier distributions cause auxin to move in a rootward direction within the stele, redistribute at the root tip, and move in a shootward direction through the root's outer layers; these auxin dynamics are essential for controlling root phenotype (Blilou et al., 2005; Swarup et al., 2005; Grieneisen et al., 2007; Lewis et al., 2007; Benjamins and Scheres, 2008; Spalding, 2013; Band et al., 2014). Changes in the expression, localisation or activity of the carriers enables the auxin distribution to be regulated by other hormones and by environmental inputs, providing a key method both for hormone crosstalk and to enable developmental responses to environmental conditions such as nutrient or water status (Kazan, 2013).

Numerous computational models have focused on how carrier-mediated auxin transport leads to the root-tip auxin dynamics (Swarup et al., 2005; Grieneisen et al., 2007; Jones et al., 2009; Band et al., 2014; Van den Berg et al., 2016; Xuan et al., 2016; Di Mambro et al., 2017) and have provided understanding of lateral root initiation (Xuan et al., 2016), gravitropism (Swarup et al., 2005), root hair growth (Jones et al., 2009) and halotropism (Van den Berg et al., 2016). We recently developed a vertex-based model of auxin transport within a real multicellular root-tip geometry (Band et al., 2014; Mellor et al., 2016; Xuan et al., 2016). The model revealed that the AUX1/LAX influx carriers control which tissues have high auxin levels, whereas PIN efflux carriers control the direction of auxin transport within these tissues (Band et al., 2014).

In addition to auxin transport, local auxin metabolism also influences the root-tip auxin distribution and the resulting root phenotypes, and can be regulated by other hormones and environmental conditions (Ljung, 2013; Korver et al., 2018). Key auxin synthesis and conversion enzymes have been shown to be cell type specific (Stepanova et al., 2008; Xuan et al., 2015) and influence auxin patterning (Brumos et al., 2018). However, the auxin metabolism network is complex, with multiple parallel pathways and feedback loops that enable auxin homeostasis (Ljung, 2013; Korasick et al., 2013; Porco et al., 2016), and an initial computational model incorporating the details of the auxin metabolism network found that perturbing key auxin degradation genes resulted in only a small modification to the auxin pattern created by the transport components, suggesting that local degradation plays a secondary role in establishing the auxin pattern (Mellor et al., 2016).

In this study, we first test our previously published auxin-transport model (Band et al., 2014) quantitatively against experimental data using the nuclear yellow fluorescent protein (YFP) auxin sensor DII-VENUS, which is an Aux/IAA-based reporter composed of a constitutively expressed fusion of the auxin-binding domain (DII) of the Aux/IAA28 protein to a fast-maturating variant of YFP, VENUS (Brunoud et al., 2012). In order to degrade DII-VENUS, auxin first binds to the TIR1/AFB receptors, and the resulting complex can then bind with DII-VENUS to promote its degradation. Thus, in addition to auxin, DII-VENUS fluorescence depends on the levels of TIR1/AFB co-receptors and the expression of the 35S promoter; however, these have both been shown to be relatively uniform within the root tip, and so the relationship between DII-VENUS nuclear fluorescence and auxin concentration can be represented by a small interaction network (shown in Fig. S1), (Brunoud et al., 2012; Band et al., 2012). We previously developed and parameterised a mechanistic model of the network of interactions through which auxin promotes DII-VENUS degradation (Band et al., 2012). In this study, we use this parameterised network model to predict DII-VENUS levels within each cell. We quantify the difference between our model predictions and experimental DII-VENUS data in wild type as well as *pin2* and *aux1* mutant backgrounds and found significant differences between the predicted and observed DII-VENUS distributions.

We hypothesised that the discrepancy between the predicted and observed DII-VENUS distributions could be caused by the presence of an additional transport component such as diffusion through plasmodesmata, which are narrow pores directly linking the cytoplasm of adjacent plant cells (Sevilem et al., 2013). One study reported fluxes of small molecules (such as auxin) through plasmodesmata within root tissues (Rutschow et al., 2011), and genetic manipulation of plasmodesmata has been shown to affect auxin dynamics during lateral root formation (Benitez-Alfonso et al., 2013), shoot tropisms (Han et al., 2014) and stem cell niche maintenance (Liu et al., 2017b; Han et al., 2019). Despite these experimental studies, passive auxin diffusion through plasmodesmata has not been included in previous auxin-transport models. We report the functional importance of plasmodesmatal diffusion, combined with carrier-mediated fluxes, in creating the root-tip auxin distribution.

## RESULTS

### Carrier-mediated transport does not explain root-tip auxin distribution

**Fig. 1.**
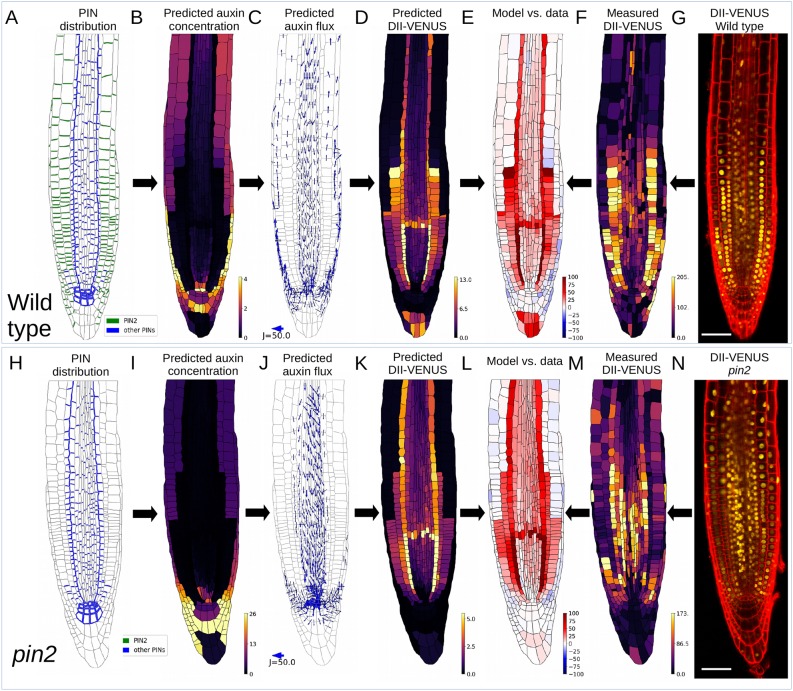
**Root tip-auxin distribution cannot be accounted for by carrier-mediated transport alone.** (A-N) Model predictions with no plasmodesmata in wild type (B-E) and *pin2* (I-L). (A,H) Prescribed PIN distribution. (B,I) Predicted steady-state auxin distribution. (C,J) Predicted auxin fluxes. Arrow width and length are proportional to flux (see scale). Only fluxes greater than 0.5 µm^−2^ s^−1^ are shown. (D,K) Predicted DII-VENUS distribution. (E,L) Difference between normalised predicted and observed DII-VENUS distribution (from predictions in D,K and data in F,M). (F,M) Quantified DII-VENUS distributions from images in G,N. (G,N) Representative DII-VENUS confocal images. Scale bars: 50 µm. See Fig. S7 for replicates.

We first simulated a model of carrier-mediated auxin transport and auxin-mediated DII-VENUS degradation (as developed by Band et al., 2014). Cell geometries, connectivities and nuclear fluorescence were segmented from confocal images of propidium iodide-stained root tips from DII-VENUS reporter lines using the SurfaceProject and CellSeT image segmentation tools (Pound et al., 2012; Band et al., 2014). We used CellSeT to manually assign a cell type to each cell and then read the geometrical and cell-type data into a tissue database (based on the OpenAlea tissue structure; Pradal et al., 2008). Distributions of AUX1, LAX and PIN carriers were automatically specified on these root-tip templates using rules developed by Band et al. (2014) based on our observations using anti-PIN antibodies (shown in figure S3 of Band et al., 2014) and data in the literature (Friml et al., 2002a,b; Blilou et al., 2005; Abas et al., 2006; Müller et al., 1998; Swarup et al., 2005, 2008; Péret et al., 2012) (Fig. 1A, Figs S2, S3; Materials and Methods). In addition, weak background efflux carriers were included on all cell membranes to account for non-polar PINs and the ABCB transporters. We specified low auxin production and degradation rates within every cell and higher auxin production in the quiescent centre (QC) and columella initials and lateral root cap (LRC), reflecting the observed distributions of auxin biosynthesis enzymes (Stepanova et al., 2008) and IBA-IAA conversion enzymes (Xuan et al., 2015).

With these data and model assumptions, we generated a system of linear ordinary differential equations (ODEs) for the auxin concentration within each cell and cell-wall compartment, which contain terms representing passive diffusion of protonated auxin across cell membranes, carrier-mediated transport of anionic auxin across cell membranes, passive auxin diffusion within the cell wall and auxin synthesis and degradation. This system of ODEs was augmented by ODEs representing the network of interactions through which auxin degrades DII-VENUS (Fig. S1), which were previously derived and parameterised using auxin-dose-response data in Band et al. (2012). Using parameter values as suggested in the literature (summarised in Table S2) the steady state of the system of ODEs was computed directly using a linear system solver in Python to give the predicted root-tip auxin and DII-VENUS distributions. See Materials and Methods and supplementary Materials and Methods for further details.

As in our previous study (Band et al., 2014) the model predicted that, in wild type, auxin levels are high within the QC region, the LRC and the elongation-zone epidermis and cortex (Fig. 1B). Owing to PIN polarity, auxin moves in a rootward direction within the stele and a shootward direction in the outer tissues (Fig. 1C). To compare the predicted and observed DII-VENUS distributions quantitatively (Fig. 1D,F,G), we calculated the normalised difference for each cell (normalising the values in each case to the minimum value for that case) (Fig. 1E). There appeared to be a far greater contrast between regions of high and low DII-VENUS concentration in the model predictions than in the data, with the predicted DII-VENUS in the meristematic cells underlying the LRC being higher than that observed.

We also quantitatively compared the predicted and observed DII-VENUS distributions in an *aux1* knockout mutant. The model predictions (Fig. S4A-D) showed that the AUX1 influx carriers have a significant effect on the auxin distribution and appear to determine which tissues have high auxin (compare Fig. 1B,C and Fig.  S4B,C); however, the predicted DII-VENUS distribution again showed greater contrast than was observed experimentally (Figs S4C-F and S5).

To test the model further, we considered the role of PIN2, which is a key contributor to shootward auxin transport (Rashotte et al., 2000) and gravitropic bending (Luschnig et al., 1998; Chen et al., 1998; Müller et al., 1998). Removing PIN2 from our model (using the wild-type root-tip template), we predicted negligible shootward auxin fluxes through the root's outer layers, as expected (Fig. S6A). However, with PIN2 removed, the predicted auxin distribution (Fig. S6A,B) is similar to that observed in wild type except in the columella region where we predict higher auxin concentrations in *pin2* (compared with wild type; Fig. 1B), owing to the absence of the shootward auxin flux away from this region in *pin2*. This finding, that it is predominantly the flux (but not the concentration) pattern that is perturbed in *pin2*, is consistent with our previous suggestion that AUX1 and LAX determine the tissues with high auxin whereas PINs mediate the fluxes within these tissues (Band et al., 2014). To test this model prediction, we created a new line by crossing the *pin2* knockout allele with the DII-VENUS sensor (Fig. 1N, Fig S7). Using the corresponding root-tip template and removing PIN2 from our simulations (Fig. 1H), we again predicted that in *pin2* mutants the shootward auxin fluxes through the root's outer layers are negligible (Fig. 1J) and the predicted auxin and DII-VENUS distributions (Fig. 1I,K) are similar to those of wild type (Fig. 1B,D).

Although the model predicted similar DII-VENUS distributions in wild type and *pin2*, quantification of the observed DII-VENUS levels in *pin2* (Fig. 1M,N) revealed key differences between the observed DII-VENUS distribution in *pin2* and wild type. In particular, we observed that DII-VENUS levels in the elongation-zone epidermis and cortex are relatively high in *pin2* (compared with the wild type) (Fig. 1M), suggesting that the removal of PIN2 reduces auxin levels in the root's outer layers (Fig. 1K). As a result, the model predictions for DII-VENUS *pin2* are not in good agreement with the data (Fig. 1L).

Previous experimental studies have shown that in the *pin2* mutant, PIN1 is ectopically expressed and localised on the shootward-facing membranes within the meristem, following the PIN2 expression pattern, thus partially restoring the wild-type PIN efflux carrier pattern (Vieten et al., 2005; Omelyanchuk et al., 2016). To test how this affects our model predictions, we introduced ectopic PIN1 into our *pin2* model using the localisation data given in Omelyanchuk et al. (2016). We found only minor differences between the *pin2* model predictions with and without ectopic PIN1 (Fig. S8), with slightly less auxin in the division zone and slightly more in the elongation zone with ectopic PIN1, suggesting that the observed ectopic PIN1 is not sufficient to restore the wild-type auxin dynamics in the *pin2* mutant.

We initially hypothesised that increasing the level of the non-polar background efflux (which represents the auxin fluxes mediated by the non-polar PIN and ABCB membrane proteins) might enable auxin to leave the outer AUX1-expressing cells to move to the inner cell layers, which would improve agreement between the predicted and observed DII-VENUS distributions. To test this hypothesis, we increased the non-polar background permeability, but found only a small improvement in the agreement between predicted and observed DII-VENUS distributions in both wild type and *pin2* (Fig. S9).

We also considered whether changing the values of the permeabilities associated with the carriers would improve the agreement between the predictions and data, as the values of these parameters have not been well characterised (Kramer et al., 2011; Rutschow et al., 2014). To test the effect of these parameters on our model predictions, we ran simulations, for both wild type and *pin2*, using permeability parameter values set to both half and double their original value for PIN efflux (Fig. S10), AUX1 influx (Fig. S11) and LAX influx (Fig. S12). In each case, we predicted minor differences in DII-VENUS levels; however, the overall pattern remains the same. In all cases considered, DII-VENUS was predicted to be low in the elongating epidermis and cortex in *pin2*, in contrast to the data, and we therefore concluded that changing the permeability parameter values does not enable the model to agree with the data.

We concluded that our current model is unable to reproduce the DII-VENUS data and that carrier-mediated auxin transport alone does not appear to account for root-tip auxin distribution.

### Incorporating plasmodesmata improves agreement between the model and experimental data

Experimental studies have detected significant plasmodesmatal fluxes within the root tip (Rutschow et al., 2011) and have shown that plasmodesmatal auxin fluxes affect lateral root development (Benitez-Alfonso et al., 2013), shoot tropisms (Han et al., 2014) and stem cell niche maintenance (Liu et al., 2017b; Han et al., 2019). We therefore hypothesised that the lack of intercellular plasmodesmata in our model caused the discrepancy between predicted and observed DII-VENUS distributions (Fig. 1E,L). To test this idea, we introduced plasmodesmata into our multicellular root-tip auxin model.

**Fig. 2.**
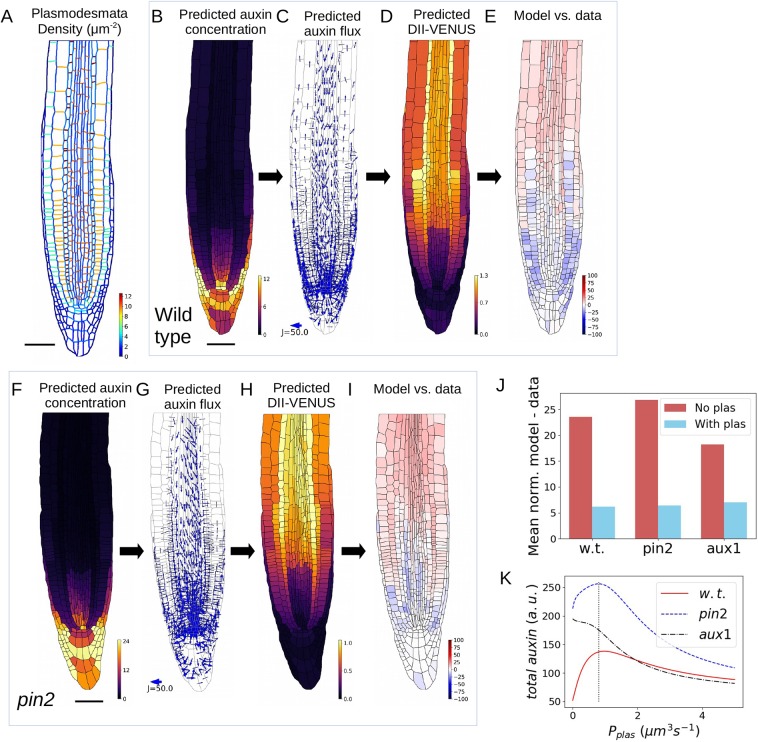
**Adding spatially variable plasmodesmatal fluxes to the model improves agreement with the data and increases overall predicted auxin in the root tip.** (A) Prescribed plasmodesmata distribution (using data from Zhu et al., 1998). (B-I) Model predictions including plasmodesmata in wild type (B-E) and *pin2* (F-I). (B,F) Predicted steady-state auxin distribution. (C,G) Predicted auxin fluxes (settings as in Fig. 1C). (D,H) Predicted DII-VENUS distribution. (E,I) Difference between normalised predicted and observed DII-VENUS distribution (from predictions in D,H and data in Fig. 1F,M). (J) Quantification of the difference between the predicted and observed DII-VENUS distribution with and without plasmodesmata. The bars show the mean absolute differences between the normalised predictions and data for every cell in wild type (w.t.), *pin2* and *aux1.* (K) Effect of plasmodesmatal permeability on the predicted total root-tip auxin in wild type, *pin2* and *aux1* (i.e. the total number of auxin molecules in the root tip; see supplementary Materials and Methods, section 2.5)*.* Dotted line shows the value of P_plas_ estimated using data from Zhu et al. (1998) and Rutschow et al. (2011). a.u., arbitrary unit. Scale bars: 50 µm.

Plasmodesmata enable passive auxin diffusion between the symplast of adjacent cells, thus enabling auxin fluxes from cells of high concentration to those of low concentration. We introduced this into the model by incorporating additional terms to the system of ODEs, prescribing the plasmodesmatal auxin flux between adjacent cells to be proportional to the concentration difference in auxin between each cell's cytoplasm. In these plasmodesmatal flux terms, the proportionality constant is equal to the (spatially variable) density of plasmodesmata multiplied by a constant permeability per plasmodesmata, P_plas_. Between adjacent cells, we specified the plasmodesmatal density using the detailed electron microscopy data of Zhu et al. (1998), who showed how plasmodesmatal density depends on cell type and position (see Fig. 2A and Table S1). These data suggest that the plasmodesmatal density is high between adjacent cells in each tissue layer (5.42-12.58 plasmodesmata per µm^2^), but low between adjacent cells of different tissue layers (2.33-3.08 plasmodesmata per µm^2^). We estimated a value of P_plas_=0.8 μm^3^ s^−1^ by dividing the permeability of 8.0 μm s^−1^ recorded by Rutschow et al. (2011) in the *Arabidopsis* root stele by the plasmodesmatal density of 9.92 μm^−2^ measured in the same tissue by Zhu et al. (1998). Note that although the model template is strictly speaking only two-dimensional, we assume the walls have unit depth in order to retain and use the experimental parameters in their original units, resulting in our estimate for the permeability per plasmodesmata, P_plas_, having units of μm^3^ s^−1^ (see supplementary Materials and Methods for further details).

We found that introducing plasmodesmatal auxin fluxes improved the overall agreement between the predicted and observed DII-VENUS distributions in wild type, *pin2* and *aux1* (Fig. 2B-I, Fig. S4). In wild type and *pin2*, plasmodesmata allow auxin to diffuse from the cells in the outer root tissue layers, where auxin concentrations are high, into the inner tissue layers, where auxin concentrations are lower. Thus, the presence of plasmodesmata increases the predicted auxin concentration in the meristematic epidermis and cortex underlying the LRC, for example, in agreement with the observed DII-VENUS distributions (Fig. 2B-I). Furthermore, the model predicts that in *pin2*, plasmodesmatal fluxes enable auxin to diffuse from both the LRC cells and the elongation-zone epidermal and cortical cells into the underlying tissues, bypassing the effect of AUX1 pulling apoplastic auxin back into the outer layers and resulting in lower auxin in the LRC and elongation-zone epidermis and cortex (Fig. 2F-I).

We also checked whether ectopic PIN1 in *pin2* would affect our conclusions (using the localisation data given in Omelyanchuk et al., 2016); we found that with plasmodesmata the model predictions for the *pin2* mutant with and without ectopic PIN1 are very similar (Fig. S13) and that the introduction of plasmodesmata improves agreement with the DII-VENUS experimental data in both cases (compare Fig. S8 and Fig. S13).

To compare further the predicted and observed DII-VENUS distributions, we introduced a metric: the mean of the normalised difference between the predicted and observed DII-VENUS levels in each cell. Calculating this metric for the models with and without plasmodesmata further confirmed that plasmodesmata improve agreement between model and data for wild type, *aux1* and *pin2* (Fig. 2J).

Although values for the model parameters have been suggested in the literature (as summarised in Table S2), many of these are not known precisely; in particular, the auxin biosynthesis and degradation rates and the permeabilities associated with each of the membrane proteins have not been well characterised. We therefore performed a parameter survey to assess whether the values of each of these parameters affect our results and conclusions. We found that including plasmodesmatal auxin fluxes improves agreement between the model predictions and data for wild type, *aux1* and *pin2* for wide ranges of each of these parameter values (Figs S14-S16).

To test the role of plasmodesmata further, we also considered seedlings treated with the auxin efflux inhibitor naphthylphthalamic acid (NPA) (Teale and Palme, 2018). Interestingly, although we observed the overall intensity of DII-VENUS to be weaker in the treated roots (compared with the untreated ones), the spatial pattern is maintained (Figs S7, S17). Simulating NPA treatment in our model required us to reduce both PIN permeability and the background permeability by a fixed (although unknown) amount. Our model without plasmodesmata predicted that reducing the PIN and background permeabilities makes little difference to either the peak level of DII-VENUS or the spatial pattern until the reduction in permeability is at or near 100% (effectively eliminating efflux entirely) (Fig. S18A), whereas the model predictions with plasmodesmata show a gradual reduction in both the peak level of DII-VENUS and in the sharpness of the spatial pattern (Fig. S18B). Using our fitness measure described above, we see that whatever the true level of reduction in auxi2n efflux efficacy following our NPA treatment, the model with the plasmodesmata is an improvement on the model without plasmodesmata (Fig. S18C).

In summary, we found that introducing plasmodesmatal auxin fluxes improves agreement between the model predictions and data in all cases considered. We concluded that plasmodesmatal auxin fluxes are essential to re-capitulate the experimentally derived auxin distribution.

### Plasmodesmatal auxin fluxes enable auxin reflux

The model revealed that the presence of plasmodesmata enables auxin to diffuse passively from the shootward auxin streams through the root's outer layers (where auxin concentrations are high) to the rootward auxin streams in the inner layers (Fig. 2C). For example, the model predicted that plasmodesmata enable auxin diffusion from the LRC to the underlying epidermis and cortex; thus, with plasmodesmata, the auxin concentration within the epidermis and cortex under the LRC are larger (Fig. 2B) (in contrast to the predictions without plasmodesmata; Fig. 1B) and the polar PINs in these inner layers create rootward auxin fluxes towards the QC (Fig. 2C). The importance of such an auxin reflux loop between the outer and inner root-tissue layers was proposed by Grieneisen et al. (2007) who considered the role of the PIN distribution with uniform AUX1 influx carriers. When we incorporated the AUX1 and LAX distribution in our previous study (Band et al., 2014), we found that AUX1 prevents auxin flux from the outer layers to the inner layers. We now find that, by allowing auxin to diffuse between the shootward and rootward PIN streams without entering the apoplast (where AUX1 determines the main influx direction), plasmodesmata enable the reflux loop.

We observed that the model appears to predict that the root-tip auxin concentrations are higher when plasmodesmata are included (Figs 1B and 2B). To test this quantitatively, we calculated the predicted total auxin in the root tip (i.e. representing the total number of auxin molecules) for increasing values of the plasmodesmatal permeability, P_plas_ (Fig. 2K). For all values of P_plas_, the predicted total auxin is higher in *pin2* than in wild type, owing to the reduced shootward fluxes when PIN2 is removed. For both wild type and *pin2*, the predicted total root-tip auxin increases with P_plas_ for values of P_plas_ between zero and 0.8 µm^3^ s^−1^. This is consistent with plasmodesmata facilitating reflux from the shootward streams in the outer tissues towards the rootward streams within the inner tissues, and hence enabling auxin to be retained within the root tip. For higher values of P_plas_, above 0.8 µm^3^ s^−1^, auxin distribution becomes more uniform because auxin can no longer accumulate in the AUX1- and LAX-expressing cells (Fig. S19), so as a result the total auxin in the root tip decreases.

### Differences in plasmodesmatal density are essential to predict the experimentally derived auxin distribution

**Fig. 3.**
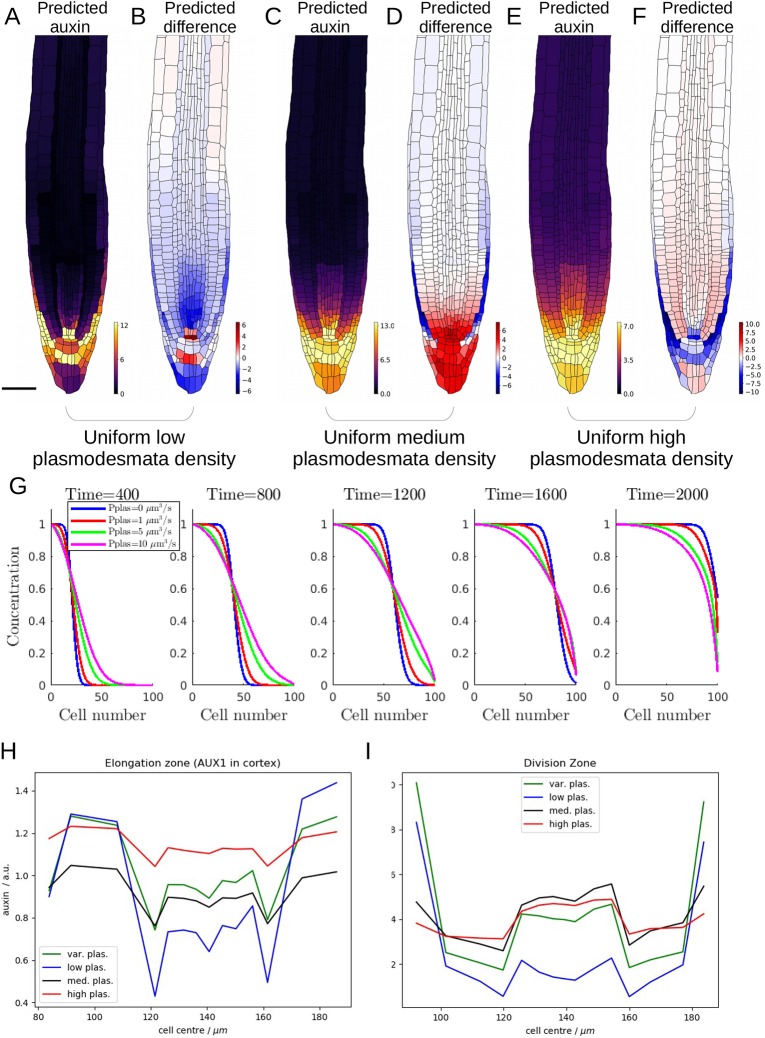
**Root tip auxin distribution is dependent on spatial variation in plasmodesmatal density, which reduces the gradient between regions of low and high auxin in a tissue-specific manner.** (A,C,E) Predicted steady-state auxin distribution with uniform plasmodesmatal density at three increasing densities; (B,D,F) Difference between the predicted auxin concentrations for the uniform plasmodesmata model (shown in A,C,E) and the variable plasmodesmata model (shown in Fig. 2B). (A,B) Low plasmodesmatal density (0.83 µm^−2^ as in periclinal walls between lateral root cap and epidermis). (C,D) Medium plasmodesmatal density (5.42 µm^−2^ as in anticlinal epidermal walls). (E,F) High plasmodesmatal density (12.58 µm^−2^ as in anticlinal endodermal walls). Scale bar: 50 µm. (G) Effect of plasmodesmata on auxin propagation through a single file of cells. We suppose that auxin moves across cell membranes via both passive diffusion of protonated auxin and active transport of anionic auxin mediated by PINs with a polar location on the downstream membrane face of each cell. We suppose that auxin also passively diffuses between adjacent cell cytoplasms through plasmodesmata. See supplementary Materials and Methods, section 2.6 for the model equations. (H,I) Horizontal profile across the root radius of the cytoplasmic auxin concentrations in the region of the elongation zone where AUX1 is expressed (H) and the (lower) division zone for the variable plasmodesmata model (I), and the models with uniformly low, medium and high plasmodesmatal densities (as defined above).

We next assessed whether the spatially variable plasmodesmatal densities (shown in Fig. 2A) are important for the wild-type auxin distribution. As one would expect, with very low uniform plasmodesmatal densities, the predicted auxin distribution is closer to the model without plasmodesmata than the spatially variable plasmodesmatal model (Fig. 3A,B). At intermediate uniform plasmodesmatal densities, the model predicts increased auxin concentration throughout the columella, suggesting that the observed low plasmodesmatal densities in this area are essential for the differences in auxin concentration between the columella tiers (Fig. 3C,D). Specifying high, uniform plasmodesmatal densities (which predominantly increases the plasmodesmatal density between cells of different tissue layers), we find that plasmodesmatal diffusion over-rides the distinctive auxin pattern created by the PIN and AUX1/LAX carriers and that the predicted auxin concentrations are more uniform across the different tissue layers (Fig. 3E,F,H,I, Fig S20). Similar effects are observed in the *pin2* and *aux1* models (Fig. S21). We concluded that differences in plasmodesmatal density (as in Fig. 2A) are essential to predict the experimentally derived auxin distribution.

To assess how plasmodesmatal density affects the auxin fluxes through the individual tissue layers, we simulated the auxin dynamics through a file of single cells, with polar PIN carriers on the downstream cell membranes (see supplementary Materials and Methods section 2.6 for the model equations). With PIN carriers alone, when the upstream auxin concentration is increased, a wave of this higher auxin concentration propagates through the cell file (Fig. 3G, blue line). Introducing plasmodesmata, we find plasmodesmatal auxin fluxes contribute a diffusive component that modifies the propagation of the wave front (Fig. 3G, Fig S22). These single-file simulations revealed that the high plasmodesmatal density between adjacent cells within each tissue layer enables plasmodesmatal diffusion to modify the shootward and rootward auxin fluxes created by the PIN carriers (without affecting the effective PIN-mediated auxin velocity through the cell file).

### Manipulating plasmodesmatal permeability alters root auxin distribution

**Fig. 4.**
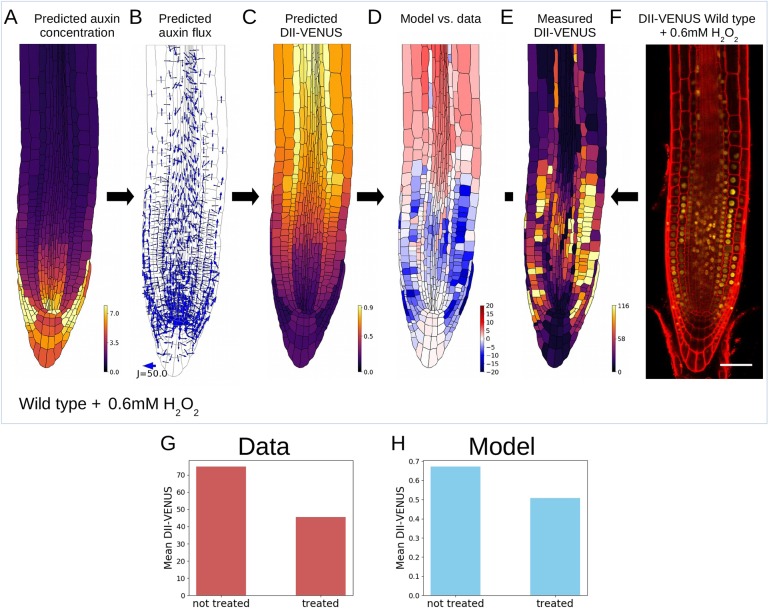
**Increasing plasmodesmatal permeability experimentally using H_2_O_2_ treatment produces results consistent with our model, increasing overall auxin concentrations.** Experimental perturbations of plasmodesmatal permeability using a 2 h treatment with 0.6 mM H_2_O_2_; in the model simulations, the H_2_O_2_ treatment was represented by doubling the value of P_plas_ to 1.6 µm^3^ s^−1^ (Rutschow et al. 2011). (A) Predicted steady-state auxin distribution. (B) Predicted auxin fluxes (settings as in Fig. 1C). (C) Predicted DII-VENUS distribution. (D) Difference between normalised predicted and observed DII-VENUS distribution (from the prediction in C and data in E). (E) Quantified DII-VENUS distribution from the image in F. (F) Representative DII-VENUS confocal image of wild-type root following a 2 h treatment with 0.6 mM H_2_O_2_. Scale bar: 50 µm. See Fig. S23 for replicates. (G) Comparison of mean cellular DII-VENUS between untreated (Fig. 1F) and 0.6 mM H_2_O_2_-treated (E) root data. (H) Comparison of mean cellular DII-VENUS between untreated (Fig. 2D) and 0.6 mM H_2_O_2_ treated (C) model predictions.

To test the model predictions that plasmodesmata significantly affect auxin distribution, we experimentally perturbed plasmodesmatal permeability. We first considered a treatment with H_2_O_2_, choosing a treatment time of 2 h and concentration of 0.6 mM because previously this has been shown to double plasmodesmatal permeabilities within the root (Rutschow et al., 2011) and longer treatments have been shown to affect carrier expression levels (Su et al., 2016). Doubling the plasmodesmatal permeability in the model, we predicted the auxin (and corresponding DII-VENUS) distribution to be much more uniform (compare Figs 4A-C and 2B-D); directly testing this prediction by applying a 0.6 mM H_2_O_2_ treatment to a wild-type DII-VENUS root tip revealed close agreement with model predictions (Fig. 4C-F). Furthermore, both predicted and observed DII-VENUS levels are reduced with the H_2_O_2_ treatment (Fig. 4G,H).

We also applied the 0.6 mM H_2_O_2_ treatment to DII-VENUS *pin2* and *aux1* lines. We found that in the treated cases both the predicted and observed distributions are more uniform in *pin2* (Fig. S24A-F), whereas in *aux1* (where the auxin distribution is already approximately uniform in the meristem and elongation zone) there appears to be a less well-defined auxin maximum around the QC (Fig. S24G-L).

We next genetically manipulated plasmodesmatal permeability. Plasmodesmatal permeability is modified by callose deposition (Chen and Kim, 2009; Rutschow et al., 2011), and key regulators include GLUCAN SYNTHASE LIKE 8 (GSL8) and CALLOSE SYNTHASE 3 (CALS3), which regulate callose synthesis (Vatén et al., 2011; Han et al., 2014); b-1,3-glucanase, which contributes to callose turnover; and PLASMODESMATA CALLOSE BINDING1 (PDCB1), which binds callose in the apoplast around the plasmodesmata (Simpson et al., 2009).

**Fig. 5.**
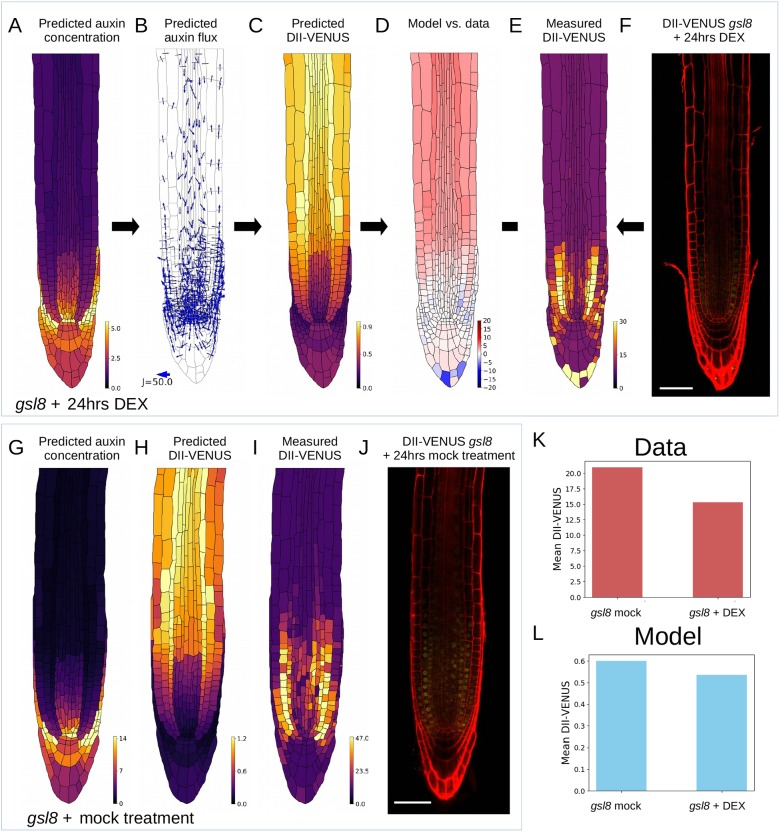
**An experimentally inducible knockout of plasmodesmatal callose deposition via the *GSL8* gene results in lowered DII-VENUS, consistent with the increased auxin predicted by the model due to elevated plasmodesmatal permeability.** Experimental perturbations of plasmodesmatal permeability via genetic manipulation using DEX-inducible *gsl8*; in the model simulations, DEX treatment was represented by doubling the value of P_plas_ to 1.6 µm^3^ s^−1^. (A-F) *gsl8*-induced model predictions and data. (G-J) Mock-treated *gsl8* model predictions and data. (A) Predicted steady-state auxin distribution. (B) Predicted auxin fluxes (settings as in Fig. 1C). (C) Predicted DII-VENUS distribution. (D) Difference between normalised predicted and observed DII-VENUS distribution (from the prediction in C and data in E). (E) Quantified DII-VENUS distribution from the image in F. (F) Representative DII-VENUS confocal image of *gsl8* root after 24 h DEX treatment. (G) Predicted steady-state auxin. (H) Predicted DII-VENUS distribution. (I) Quantified DII-VENUS distribution from the image in J. (J) Representative DII-VENUS confocal image of mock-treated *gsl8* root. See Fig. S26 for replicates of both DEX-treated roots and mock-treated control roots. (K) Comparison of mean cellular DII-VENUS between mock-treated (I) and DEX-treated (E) *gsl8* root data. (L) Comparison of mean cellular DII-VENUS between mock-treated (H) and DEX-treated (C) model predictions. Scale bars: 50 µm.

To assess the role of plasmodesmata further, we genetically manipulated plasmodesmatal permeability by lowering levels of *GSL8*, which is highly expressed in root tips (Chen and Kim, 2009) and has been shown to increase plasmodesmatal diffusion permeability in the shoot (Han et al., 2014). Given that *GSL8* has been shown to be induced by auxin in the shoot (Han et al., 2014), we first checked whether a similar response is present in the root, using an auxin treatment root transcriptomics data set (Voß et al., 2015). In contrast to the findings in the shoot (Han et al., 2014), these root-specific data revealed no differences in the expression of *GSL8* (or of b-1,3-glucanase or PDCB1) after an auxin dose, suggesting that plasmodesmatal permeability does not appear to be affected by auxin levels in the root (Fig. S25). We therefore employed a dexamethasone (DEX)-inducible GSL8 anti-miRNA DII-VENUS line to simultaneously downregulate callose deposition and monitor auxin levels. We observed a more uniform DII-VENUS distribution, which is in good agreement with the model predictions (in which we represented the lower GSL8 by doubling plasmodesmatal permeabilities) (Fig. 5A-F, Fig. S26). In contrast, the mock-treated control *gsl8* root (in which GSL8 is still expressed) exhibits higher DII-VENUS, both experimentally and in the model, than the DEX treated root (Fig. 5G-L). We concluded that manipulating plasmodesmatal permeability significantly modifies root-tip auxin distribution.

## DISCUSSION

The auxin distribution in the root tip controls many aspects of root phenotype. Previous studies have uncovered how carrier-mediated auxin transport creates distinctive auxin distribution and fluxes (Swarup et al., 2005; Grieneisen et al., 2007; Jones et al., 2009; Band et al., 2014; Xuan et al., 2016; Van den Berg et al., 2016; Di Mambro et al., 2017); however, passive auxin diffusion through plasmodesmata has not been included in previous computational models and its effect on the root-tip auxin distribution has not been considered. Using a systems approach, we demonstrated that auxin diffusion through plasmodesmata has a major impact on the root-tip auxin distribution. Although our previous model suggested that the AUX1/LAX influx carriers control which tissues have high auxin levels (Band et al., 2014), quantitative comparison between the model predictions and experimental data showed that carrier-mediated auxin transport alone does not explain the root-tip auxin distribution (as observed using the DII-VENUS auxin sensor). However, introducing passive auxin fluxes through plasmodesmata improved the agreement between the predicted and observed DII-VENUS distributions.

A modelling approach enabled us to characterise not only the auxin distribution (which could be inferred experimentally from DII-VENUS images), but also predict the flux pattern within the root tip, which cannot be detected directly experimentally. We found that plasmodesmata enable fluxes between adjacent tissue layers by allowing auxin to move between the transport streams created by the polar PIN proteins. Plasmodesmatal auxin fluxes thus create a reflux loop (as proposed by Grieneisen et al., 2007) and increase the predicted amount of auxin within the root tip. The reflux into the inner layers significantly increases the auxin concentration within the meristematic tissues underlying the LRC; given that auxin within each tissue is thought to control meristem size (Di Mambro et al., 2017) and affect gravitropism (Rahman et al., 2010), our new model with substantial auxin within the meristem is consistent with these observations. Given that auxin distribution affects a wide range of processes within the root-tip, the modified auxin distribution is likely to impact our understanding of numerous auxin-related phenotypes.

**Fig. 6.**
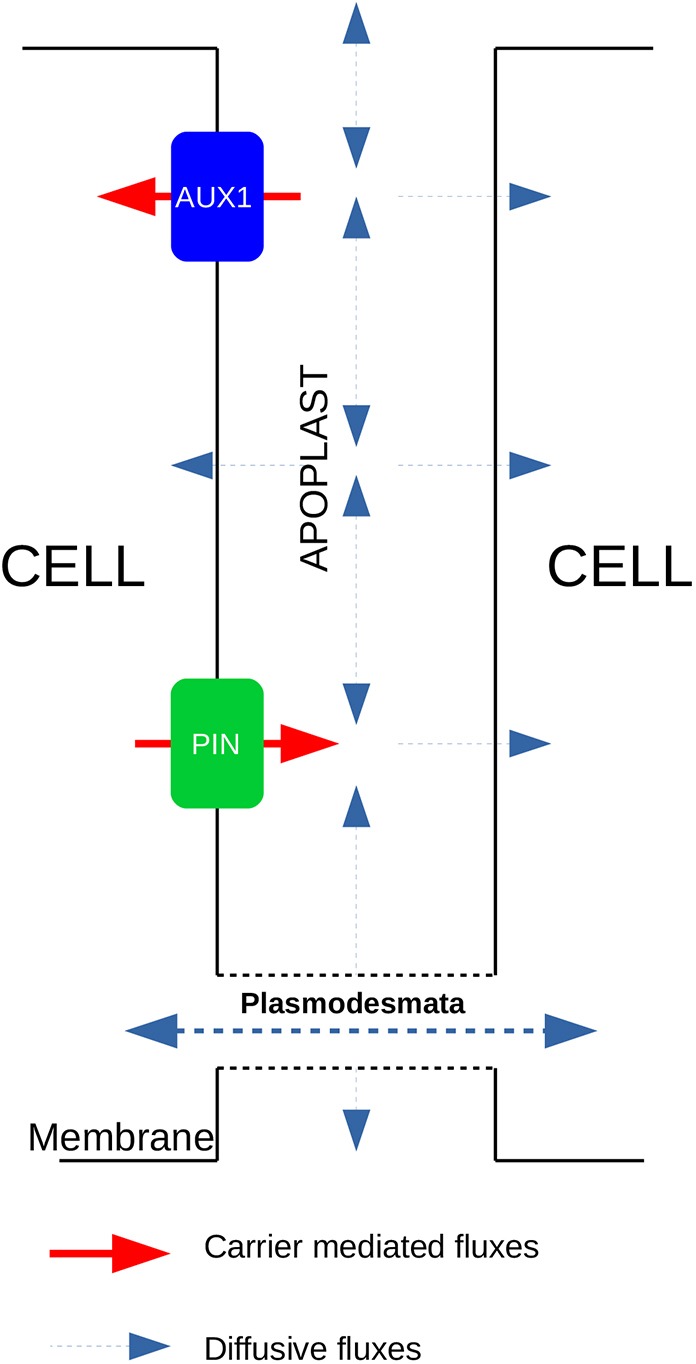
**Symplastic fluxes of auxin via the apoplast can bypass carrier-mediated auxin fluxes.** Schematic of the auxin fluxes in the model. Although auxin transported via membrane-bound carriers must travel through the apoplast to reach adjacent cells, the presence of plasmodesmata enables diffusion of auxin directly into adjacent cells. In addition, auxin may diffuse within the apoplast itself.

Our study clearly demonstrates the importance of the symplastic pathway in establishing auxin patterns. The symplastic pathway via plasmodesmata is important in auxin redistribution because it enables auxin to bypass any transporters and move directly from cell to cell without entering the apoplast (Fig. 6). As movement through plasmodesmata is thought to be via simple diffusion, plasmodesmata enable auxin to move down concentration gradients, from cells of high auxin concentration to cells with lower auxin concentration. Although cells expressing AUX1/LAX influx carriers still accumulate more auxin than neighbouring cells without AUX1/LAX, the plasmodesmata allow auxin to diffuse into those neighbouring cells thereby reducing the concentration differences. Furthermore, we found that although the overall speed of auxin through the tissue is not affected by the presence of plasmodesmata, the gradient between adjacent cells is less sharp (Fig. 3G). Thus, regulation of plasmodesmata permeability would offer a way to fine-tune cellular auxin concentrations while maintaining the overall flux and pattern within the root tip.

As more data and knowledge become available, extending this model to incorporate further details of the auxin metabolism network, carrier regulation and hormone crosstalk would provide further insights into how plasmodesmata affect hormone-regulated root development. For example, several recent computational models have investigated how hormone crosstalk modifies the root-tip auxin pattern via regulation of both auxin transport and synthesis (Moore et al., 2015; Di Mambro et al., 2017); considering how hormone diffusion through plasmodesmata affects the predictions from hormone crosstalk models would further elucidate their role. In addition, several studies have shown that other hormones, such as ABA (Liu et al., 2017a), gibberellic acid (Rinne et al., 2011) and salicylic acid (Wang et al., 2013), regulate plasmodematal gating via regulation of callose deposition, and so as well as hormonal crosstalk on a transcriptional level, future crosstalk models may also need to consider regulation of hormonal movement via plasmodesmata.

Further characterisation of the diffusion rates through plasmodesmata would also be beneficial. Our model used detailed measurements of plasmodesmatal density and assumed that the rate of plasmodesmatal auxin diffusion is proportional to the plasmodesmatal density. However, it may be that plasmodesmatal gating alters these diffusion rates in a cell type-specific manner, and so future measurements may enable us to test this model assumption and refine future models. For instance, cell type-specific symplastic movement of small photoinducible fluorescent proteins (DRONPA) at the root tip was measured recently (Gerlitz et al., 2018) and the QC cells were found to be highly connected to the columella (although we note that the molecules used in the study were significantly larger than auxin). Furthermore, plasmodesmatal density or gating may be altered in genetic mutants such as *aux1* and *pin2*, which could be considered in future models should data become available.

In addition to auxin, plasmodesmata are thought to be conduits for many signals, such as microRNAs, transcription factors, water and nutrients (Vatén et al., 2011; Ross-Elliott et al., 2017); for example, microRNA diffusion has been shown to be essential for vasculature patterning within the root (Muraro et al., 2014). We envisage that cell-to-cell communication via these molecules will impact developmental patterning and will form an extra level of regulation in addition to hormone-regulated patterning. Regulation of fluxes through plasmodesmata via callose deposition would allow the plant to target numerous pathways to control development.

## MATERIALS AND METHODS

### Model description

Multicellular root tip geometries were obtained from the confocal images using SurfaceProject and CellSeT (Pound et al., 2012; Band et al., 2014).

These data, along with the assignation of auxin influx and efflux carriers to specific cell membranes based on neighbouring cell types, were used to generate a system of linear ODEs for the auxin concentration within each cell and cell-wall compartment and DII-VENUS within each cell. Along with small production and degradation terms in every cell, there was a fixed, non-zero boundary condition for auxin in the stele at the shootward end of the tissue, representing a constant supply of auxin from the shoot. The steady state of the ODEs was computed directly using a linear system solver in Python. See supplementary Materials and Methods for a full model definition.

The prescribed carrier distributions are shown in Fig. 1A and Fig. S3. PIN1 was specified to be on the rootward-facing membranes in the endodermis and stele. PIN2 was specified to be on the shootward-facing membranes in the LRC, elongation zone and distal-meristem epidermis and elongation-zone cortex, and on the rootward-facing membranes of the meristematic cortex. PIN3 was specified to be on the rootward-facing membranes of the stele and endodermis, the inward-facing lateral membranes of the endodermis and all faces of the columella initials and S1 and S2 tiers; PIN4 to be on rootward-facing membranes in the proximal meristematic cells of the epidermis, cortex, endodermis and stele and on all faces of cells in the QC, columella initials and S1 and S2 tiers. PIN7 was specified to be on the rootward-facing membranes in the stele. We specified AUX1 to be present in the LRC, elongation zone epidermis and cortex (with expression in the cortex first appearing in a more shootward position than in the epidermis), and S1, S2 and S3 tiers of the columella; LAX2 to be present in the QC, columella initials, and rootward half of the meristematic stele; and LAX3 to be present only in the S2 tier of the columella.

### Plant material and growth conditions

Seeds were surface sterilised with 50% (vol/vol) hypochlorous acid for 5 min and then washed three times with sterile deionised water. Plant seeds were plated on 0.5 strength Murashige and Skoog medium (2.17 g salts per 1 l), at pH 5.8 and solidified with 1% plant agar (Duchefa). Seeds were stratified at 4°C for 48 h in the dark to synchronise germination, and then incubated vertically in a culture room under 12 h light at 22°C and 12 h dark at 22°C (light: 120-150 µmol m^−2^ s^−1^). The *Arabidopsis* ecotype Columbia (Col-0) was used as the wild type in all experiments.

For manipulation of plasmodesmatal permeability, DII-VENUS lines were treated with 0.6 mM H_2_O_2_ for 2 h to open plasmodesmata, as described by Rutschow et al. (2011). In addition, we used dsGSL8 lines (Han et al., 2014) in which plasmodesmatal opening was induced with 24 h treatment of 20 µM dexamethasone. RNAi induction in all used lines was confirmed by germination on medium on 20 µM dexamethasone (as described by Han et al., 2014).

### Microscopy

Confocal microscopy was performed using a Leica SP8 confocal laser scanning microscope (Leica Microsystems). Cell walls were stained using propidium iodide (10 µg ml^−1^; Sigma-Aldrich). Scanning settings used for one experiment were optimised and kept unchanged throughout the experiments.

## Supplementary Material

Supplementary information
