# Supplementary material for: Auxin fluxes through plasmodesmata modify root-tip auxin distribution
Source: Development. 2020 Mar 30;147(6):dev181669. doi: 10.1242/dev.181669 (PMC7132777; doi:10.1242/dev.181669)
Supplement: Supplementary information [file develop-147-181669-s1.pdf]

Supplementary Materials and Methods

Mellor N., Voß U., Janes G., Bennett M.J., Wells D.M., and Band L.R.

S1 Model Definition

The multicell model shown in Figure 1 is broadly as defined in Band et al. (2014), Figure 6, Supplemental methods sections 2-6, except as follows:

- Rather than scaling the effect of multiple PINs on a given membrane as before, the effect of multiple PINs is now additive. So if, for example, a membrane has PIN1, PIN4 and PIN7 present, its permeability is effectively three times that of a membrane with just PIN2 present.
- After Xuan et al. (2016), auxin biosynthesis is increased 10-fold in the two outer lateral root cap layers, rather than just in the quiescent centre and initials in the Band et al. (2014).
- Rather than a zero auxin boundary condition in the outer cell layers (epidermis, cortex and endodermis) at the shootward boundary as in Band et al. (2014), we use a zero flux condition between these cells and their rootward neighbours as in Mellor et al. (2016), by setting their steady state values to be equal.

For a more detailed description of the model and these changes, and a description of the addition of plasmodesmata, see below.

S1.1 Spatial structure

As in Band et al. (2014) (Supplemental methods, section 1) the tissues are based on a 2-dimensional cellular structure of a cross-secion of the *Arabidopsis* root (approximately 500μm shootwards from the root tip) obtained using confocal microscopy with cell walls stained with propidium iodide (see Materials and Methods). In each case the roots had been crossed with the DII-VENUS nuclear-located yellow-fluorescent-protein auxin-response reporter (Band et al. 2012; Brunoud et al. 2012), and the software package SurfaceProject (Band et al. (2014), supplemental methods, section 1.1) was used to extract a 2D plane from each image stack with all the nuclei brought into the plane of focus.

Once the 2D image was obtained the cell segmentation software CellSet (Pound et al. 2012) was used to extract the position of all cell walls and cells, and quantify DII-VENUS nuclear fluorescence for every cell. Cell types were input manually using CellSet, according the the template shown in Figure S1.

The tissue is set up using Python code based on the Openalea framework (Pradal et al. 2008), and defined as a set of points in space representing the vertices of the tissue,

Development • Supplementary information

which are linked to wall objects, which in turn are linked to objects representing the cell compartments. Wall compartments may be shared between two cells in the tissue (which we define as 'inner' walls), or if associated with a cell at the edge of the tissue it is only associated with that one cell (and defined as an 'outer' wall). Cell membranes are represented by way of a directed graph between the cells.

Dyson et al. (2014) recently obtained measurements for cell wall thickness in various cell types and wall orientations in the *Arabidopsis* root tip. We assume a constant cell wall thickness ($\lambda = 0.14\mu m$) approximately consistent with these measurements, so that the 2D area of a given wall is equal to the length of that wall multiplied by λ. The area of the small vertex compartments are approximated as λ^2. Finally, the area of a given cell is calculated as the area of the polygon defined by the vertices bounding the walls adjacent to that cell.

PIN1,2,3,4 and 7 efflux carriers and AUX1 and LAX influx carriers are positioned on cell membranes according to a set of rules based on cell type, position and membrane orientation, as described in Band et al. (2014), Supplemental Tables 2 and 3. One such carrier localisation used in this paper is shown in Figure S2.

In the model where ectopic PIN1 is expressed in the *pin2* following the observations of Omelyanchuk et al. (2016) (Figures S7 and S9) we place PIN1 on the shootward facing membranes of the last 20 and 25 rootward epidermal and cortical cells respectively (on each side of the root).

S1.2 Model equations (auxin)

S1.2.1 Carrier mediated flux

The model is based on the ordinary differential equations defined previously by Band et al. (2014), Supplemental methods, section 2. In this previous model auxin flux is defined from cell compartments to adjacent wall compartments (and vice versa), to represent movement of auxin across cell membranes, either via influx and efflux carriers or via passive diffusion. Constants determining the directionality of the carriers and to model the acid trapping of auxin within the cytoplasm are used as defined previously Band et al. (2014).

Five possible flux carrier-mediated components exist between each wall and cell compartment: passive (P_{IAAH}), PIN dependent (P_{PIN}), AUX1 dependent (P_{AUX1}), LAX dependent (P_{LAX}), and a background carrier mediated flux (P_{back}). P_{IAAH} and P_{back} are ubiquitous, while P_{AUX1} and P_{LAX} are both multiplied by one or zero depending on whether AUX1 and LAX are respectively present or absent for a given cell membrane. AUX1 and LAX localisation are as defined in the final model given in Band et al. (2014) Figure 6, and Figure S2, with AUX1 expressed in the cortical and epidermal cells in the elongation zone. PIN1,2,3,4 and 7 distribution is as defined in Band et al. (2014) Supplemental Table 3, and Figure S2, with P_{PIN} scaled by the total number of PINs present for a given membrane, so that e.g. a membrane having PIN1,3 and 7 has three times the flux capacity as a membrane with just PIN2. As in Band et al. (2014) we denote the flux from the kth apoplastic compartment between cells i and j to cell i as J_{ijk} and set this

Development • Supplementary information

flux as:

$$\begin{aligned}\mathrm{J_{ijk}} =& P_{IAAH}(A_1[\text{Auxin}]_{aijk} - B_1[\text{Auxin}]_i) + P_{AUX1}[\text{AUX1}]_{ijk}(A_2[\text{Auxin}]_{aijk} - B_2[\text{Auxin}]_i)\\ &+ P_{LAX}[\text{LAX}]_{ijk}(A_2[\text{Auxin}]_{aijk} - B_2[\text{Auxin}]_i)\\ &+ P_{PIN}[\text{PIN}]_{ijk}(A_3[\text{Auxin}]_{aijk} - B_3[\text{Auxin}]_i)\\ &+ P_{back}(A_3[\text{Auxin}]_{aijk} - B_3[\text{Auxin}]_i), \end{aligned} \tag{1}$$

where A_1, A_2, A_3, B_1, B_2 and B_3 are constants (described in Band and King (2012) and given in Table S2), P_{PIN}, P_{AUX1}, P_{LAX}, P_{IAAH} and P_{back} the permeabilities (see Table S2), $[\text{Auxin}]_i$ is the auxin concentration in cell i, and $[\text{Auxin}]_{aijk}$ the auxin concentration in apoplastic compartment ijk.

S1.2.2 Apoplastic flux

As before, to simulate auxin diffusion within the apoplast, the flux between adjacent wall compartments is simulated by considering the flux from each wall to and from two small vertex compartments representing the two ends of a given wall compartment. Denoting the flux from vertex l to apoplast ijk as J_{ijkl} we have the following:

$$\mathrm{J_{ijkl}} = \frac{2\mathrm{D_{cw}}}{\mathrm{S_{ijk}}}([\text{Auxin}]_{\mathrm{vl}} - [\text{Auxin}]_{\mathrm{aijk}}), \tag{2}$$

where D_{cw} is the diffusion coefficient of auxin in the apoplast, S_{ijk} the length of compartment ijk, and $[\text{Auxin}]_{vl}$ the auxin concentration in vertex l.

S1.2.3 Plasmodesmatal flux

In the new model presented in this paper, in addition to movement across the cell membranes and within the apoplast, we consider another flux, via intercellular plasmodesmata. Since the plasmodesmata (where present) essentially link the cytoplasm of adjacent cells, we assume these fluxes are directly between the cells themselves, without being mediated by wall compartments.

The plasmodesmatal flux from cell j to cell i (J^p_{ij}) is therefore defined as :

$$\mathrm{J^p_{ij}} = \mathrm{P_{plas}}\ \mathrm{d_{ij}}([\text{Auxin}]_{\mathrm{j}} - [\text{Auxin}]_{\mathrm{i}}) \tag{3}$$

where P_{plas} is the permeability per plasmodesmata ($\mu m^3 s^{-1}$)and d_{ij} the plasmodesmatal density (μm^{-2}) between cells i and j ($d_{ij} \equiv d_{ji}$). The plasmodesmatal density varies by cell type and wall orientation and is specified using TEM data given by Zhu et al. (1998) and reproduced in Table S1.

Rutschow et al. (2011) give an experimentally measured estimate of the plasmodesmata permeability in the Arabidopsis stele in the longitudinal direction of 8 $\mu\text{m}\,\text{sec}^{-1}$. Combining this value with the measured plasmodesmata density in the anticlinal walls of the stele of 9.92 μm^{-2} given by Zhu et al. (1998) gives an estimate for the permeability per plasmodesmata of $P_{plas} = 0.806\ \mu m^3 s^{-1}$.

S1.2.4 Production and degradation

We also include in each cell a constant, cell-type dependent auxin biosythesis rate (α_i^{HIGH} in the QC, initials and outer lateral root cap, α_i^{LOW} elsewhere, and referred to generically

Development • Supplementary information

Table S1: Plasmodesmata densities (μm^{-2}) used in the model, taken from Zhu et al. (1998). Pericycle cell layer included in stele. See Figure S1 for cell type template.

Anticlinal walls	
Stele	9.92
Epidermis	5.42
Endodermis	12.58
Cortex	9.08
Lateral root cap	2.08
Columella	3.0
Columella initials to columella	4.25
QC to Columella initials	3.08
QC to stele	3.33
CE initials to endodermis/cortex	6.33
Periclinal walls	
Stele	2.42
Stele to endodermis	3.08
Epidermis to lateral root cap	0.83
Endodermis to cortex	3.00
Cortex to epidermis	2.33
Lateral root cap	0.25
Columella	1.33
Columella initials	3.58
QC	2.0
QC to CE initials	3.33
CE initials to Lateral root cap	3.0

as α_i), and a uniform, linear degradation rate (β). The auxin biosynthesis rate is unifiorm expect for the QC and initials (as in Band et al. (2014)), and in the two outer lateral root cap layers (after Xuan et al. (2016)), where it is set to be 10-fold higher than in the remaining cell types.

S1.2.5 Combined equations

Combining the fluxes given by Equations (1)-(3) and the production and degradation terms we have the following set of ODEs:

$$\frac{d[\mathrm{Auxin}]_i}{dt} = \alpha_i - \beta[\mathrm{Auxin}]_\mathrm{i} + \frac{1}{\mathrm{R_i}} \sum_{\mathrm{j \in C_i}} \sum_{\mathrm{k=1}}^{\mathrm{N_{ij}}} \mathrm{S_{ijk}} \left(\mathrm{J_{ijk}} + \mathrm{J^p_{ij}} \right), \quad (4)$$

$$\frac{d[\mathrm{Auxin}]_{aijk}}{dt} = -\frac{1}{\lambda}(\mathrm{J_{ijk}} + \mathrm{J_{jik}}) + \frac{1}{\mathrm{S_{ijk}}} \sum_{\mathrm{m \in V_{ijk}}} \mathrm{J_{ijkm}}, \quad (5)$$

$$\frac{d[\mathrm{Auxin}]_{vk}}{dt} = -\frac{1}{\lambda} \sum_{\mathrm{ijk \in W_l}} \mathrm{J_{ijkl}}, \quad (6)$$

where $\mathrm{R_i}$ is the 2-D area of cell i, C_i denotes the set of cells adjacent to cell i, N_{ij} denotes the number of apoplast compartments between cells i and j, V_{ijk} denotes the pair of vertex compartments adjacent to apoplast compartment ijk and W_l denotes the collection of apoplast compartments, ijk, adjacent to vertex l. Since the cells are defined by a set of ordered 2-D coordinates outlining an irregular polygon we can calculate their area using a simple triangulation algorithm.

S1.2.6 Boundary and initial conditions

For cells in the stele (not including the pericycle) at the shootward boundary we assume a constant fixed supply of auxin from the shoot so there is a fixed boundary condition of auxin ($[\mathrm{Auxin}]_\mathrm{b} = 1$) in these cells. For the remaining cells at the shootward boundary (i.e. pericycle, endodermis, cortex and epidermis) we assume a zero gradient boundary condition, so that steady state auxin in these cells is equal to the value in the adjacent cell in the same cell layer; i.e. for a given outer boundary cell o with rootward neighbour n we have, at steady state:

$$[\mathrm{Auxin}]_o - [\mathrm{Auxin}]_n = 0$$

S1.3 Numerical methods

Since in this paper we only consider steady-state values, and given the system is linear we can compute the steady state directly by setting the derivatives to zero and rearranging the system in the form:

$$\mathbf{J} \times [\mathbf{Auxin}] = \mathbf{r},$$

where (if n is the total number of cell, wall and vertex compartments) $\mathbf{J}$ is the $n \times n$ matrix representing all of the combined fluxes and degradation terms, $[\mathbf{Auxin}]$ is the $n \times 1$ vector of auxin concentrations in every compartment, and $\mathbf{r}$ the $n \times 1$ vector of production rates and boundary conditions. The resulting linear system is then solved using the sparse matrix solver spsolve from the Python package Numpy.

S1.4 Model equations (DII-VENUS)

Following Band et al. (2012), the DII-VENUS dynamics within each cell can be described using a system of coupled nonlinear ordinary differential equations (ODEs) for the concentrations of auxin, $[\text{Auxin}]_i$, DII-VENUS, $[\text{VENUS}]_i$, TIR1/AFB receptors, $[\text{TIR1}]_i$, auxin-TIR1/AFB complexes, $[\text{Auxin}\cdot\text{TIR1}]_i$, and auxin-TIR1/AFB-DII-VENUS complexes, $[\text{Auxin}\cdot\text{TIR1}\cdot\text{VENUS}]_i$:

$$\frac{\mathrm{d}[\text{Auxin}]_i}{\mathrm{d}t} = k_d[\text{Auxin}\cdot\text{TIR1}]_i - k_a[\text{Auxin}]_i[\text{TIR1}]_i + \alpha_i - \beta[\text{Auxin}]_i + \frac{1}{R_i}\sum_{j\in C_i}\sum_{k=1}^{N_{ij}} S_{ijk}\left(J_{ijk} + J_{ij}^p\right), \quad (7)$$

$$\frac{\mathrm{d}[\text{TIR1}]_i}{\mathrm{d}t} = -k_a[\text{Auxin}]_i[\text{TIR1}]_i + k_d[\text{Auxin}\cdot\text{TIR1}]_i, \quad (8)$$

$$\frac{\mathrm{d}[\text{Auxin}\cdot\text{TIR1}]_i}{\mathrm{d}t} = k_a[\text{Auxin}]_i[\text{TIR1}]_i - k_d[\text{Auxin}\cdot\text{TIR1}]_i + (l_d + l_m)[\text{Auxin}\cdot\text{TIR1}\cdot\text{VENUS}]_i - l_a[\text{Auxin}\cdot\text{TIR1}]_i[\text{VENUS}]_i, \quad (9)$$

$$\frac{\mathrm{d}[\text{Auxin}\cdot\text{TIR1}\cdot\text{VENUS}]_i}{\mathrm{d}t} = l_a[\text{Auxin}\cdot\text{TIR1}]_i[\text{VENUS}]_i - (l_d + l_m)[\text{Auxin}\cdot\text{TIR1}\cdot\text{VENUS}]_i, \quad (10)$$

$$\frac{\mathrm{d}[\text{VENUS}]_i}{\mathrm{d}t} = \delta - l_a[\text{VENUS}]_i[\text{Auxin}\cdot\text{TIR1}]_i + l_d[\text{Auxin}\cdot\text{TIR1}\cdot\text{VENUS}]_i, \quad (11)$$

where $i = 1, 2, \cdots, N$ labels the cells, and the final term in (7) represents the change in auxin concentration due to fluxes across the cell membrane as defined above.

As described in detail in Band et al. (2012) , if we scale these equations and suppose that complex formation occurs rapidly (i.e. the rate constants k_a, k_d, l_a, l_d and l_m are relatively large), we can reduce the network model to a single equation for the DII-VENUS concentration:

$$\frac{\mathrm{d}[\text{VENUS}]_i}{\mathrm{dt}} = p_2\left(1 - \frac{[\text{Auxin}]_\text{i}[\text{VENUS}]_\text{i}}{p_3 + p_4[\text{Auxin}]_\text{i} + \text{p}_1[\text{Auxin}]_\text{i}[\text{VENUS}]_\text{i}}\right), \quad (12)$$

where we define the parameters p_1, p_2, p_3 and p_4 as:

$$p_1 = \frac{[\text{Auxin}\cdot\text{TIR1}\cdot\text{VENUS}]_b}{[\text{TIR1}]_\text{T}},\ p_2 = \delta/[\text{VENUS}]_b,$$

$$p_3 = \frac{[\text{TIR1}]_b}{[\text{TIR1}]_\text{T}}, \quad p_4 = \frac{[\text{Auxin}\cdot\text{TIR1}]_b}{[\text{TIR1}]_\text{T}},$$

and where the b subscript represents the steady-state value of a given variable at the stele shootward boundary (see section S1.2.6), and $[\text{TIR1}]_\text{T}$ is the total (conserved) concentration of TIR1/AFB receptors in each cell. We use the parameter values $p_3 = 0.91$, $p_4 = 0.03$ and $p_1 = 0.06$ as estimated in Band et al. (2012) and used in Band et al. (2014).

Given we are calculating the steady state auxin in every cell directly, we can set the derivative of equation (12) to zero, and rearrange to obtain the following steady-state relationship:

$$[\text{VENUS}]^*_i = \frac{\frac{p_3}{[\text{Auxin}]^*_i} + p_4}{1 - p_1}, \quad (13)$$

Development • Supplementary information

where the $*$ superscript represents the steady state values of Auxin and DII-VENUS in a given cell.

S2 Simulation cases

The default set of parameters are given in Table S2. The cases given in the paper where the parameters are altered or the model is otherwise perturbed are described in more detail below.

S2.1 Permeability per plasmodesmata

For the simulations without plasmodesmata shown in Figures 1 and S4a-d, the model is as described above, with P_{plas} set to zero, while in the remaining figures P_{plas} is set to our estimated value of 0.806 $\mu m^3 s^{-1}$ unless otherwise stated. Notable exceptions to this are in Figures 4 and 5 where both the addition of $0.6\mu M$ H_2O_2 to a wild type root and the addition of DEX to a DEX inducible *gsl8* knockout mutant are simulated by a doubling of P_{plas} to 1.612 $\mu m^3 s^{-1}$.

S2.2 Plasmodesmata density

Plasmodesmata density are set according to the values (in Table S1) obtained from Zhu et al. (1998), except in Figure 3 where the density is set uniformly to $0.83\mu m^{-2}$ (low plasmodesmatal density, measured value for periclinal walls between lateral root cap and epidermis), $5.42\mu m^{-2}$ (medium plasmodesmatal density, measured value for anticlinal epidermal walls) or $12.58\mu m^{-2}$ (high plasmodesmatal density, measured value for anticlinal endodermal walls).

S2.3 Transport mutants

The simulations of the *pin2* and *aux1* mutants are implemented by setting the level of each respective transporter to zero on all cell membranes, with all remaining model parameters unchanged. For the ectopic PIN1 in *pin2* simulations (Fig. S6,S8), based on observations from Omelyanchuk et al. (2016) we add PIN1 to the shootward membranes of the 20 most rootward epidermal cells and the rootward membranes of the 25 most rootward cortical cells on either side of the root, while knocking out PIN2 entirely as before.

S2.4 Model evaluation against experimental data

To compare model predictions for DII-VENUS with measured fluorescense we normalise each value with the minimum value in each case, then plot the difference between model and data for each cell (Figures 1e,l, 2e,i, 4d, 5d, S4d,j S14d,j). To quantify this comparison (Figure 2j, S9) we take the mean absolute difference between cells in the model and cells in the data, i.e.:

$$F = \frac{\sum_{i \in C} |\text{model}_i - \text{data}_i|}{|C|},$$

where C is the set of all cell compartments (with $|C|$ denoting the number of cells), and model_i and data_i denoting model DII-VENUS and data DII-VENUS respectively, each normalised by their minimum values.

Development • Supplementary information

S2.5 Total auxin in tissue

The total auxin in the tissue (Auxin_T) as shown in Figure 2k for varying values of P_{plas} in the different simulated genotypes is calculated as:

$$\mathrm{Auxin_T} = \sum_{\mathrm{i}\in\mathrm{C}} \mathrm{R_i}[\mathrm{Auxin}]_\mathrm{i} + \sum_{\mathrm{j}\in\mathrm{W}} \lambda \mathrm{S_j}[\mathrm{Auxin}]_\mathrm{i},$$

where C is the set of all cell compartments, W is the set of all wall compartments, $\mathrm{R_i}$ is the area of cell i, $\mathrm{S_j}$ the length of wall j and λ the cell wall width.

Table S2: Estimated parameter values with associated reference (where appropriate). Parameters without units given are dimensionless.

Permeabilities		
P_{plas}	$0.806\ \mu m^3\ s^{-1}$	Rutschow et al. (2011), Zhu et al. (1998)
P_{IAAH}	$0.56\ \mu m\ s^{-1}$	Swarup et al. (2005)
P_{PIN}	$0.56\ \mu m\ s^{-1}$	Swarup et al. (2005)
P_{AUX1}	$0.56\ \mu m\ s^{-1}$	Swarup et al. (2005)
P_{LAX}	$0.56\ \mu m\ s^{-1}$	Assumed same as P_{AUX1}
P_{back}	$0.168\ \mu m\ s^{-1}$	Assumed to be 30% of P_{PIN}
Proportionality constants		
A_1	0.240	See Band and King (2012) for
A_2	3.56	derivation of all constants
A_3	0.0.34	
B_1	0.004	
B_2	0.045	
B_3	4.67	
Apoplastic Diffusion		
D_{cw}	$32\ \mu m^2\ s^{-1}$	Kramer et al. (2007)
Cell wall thickness		
λ	$0.14\ \mu m$	Dyson et al. (2014)
Auxin degradation		
β	0.001	As in Band et al. (2014)
Auxin production		
α_i^{HIGH} (QC, initials or 2 outer LRC layers)	0.01	Xuan et al. (2016); Stepanova et al. (2008)
α_i^{LOW} (elsewhere)	0.001	As in Band et al. (2014)
DII-VENUS regulation		
p_1	0.06	Band et al. (2012)
p_3	0.91	Band et al. (2012)
p_4	0.03	Band et al. (2012)

Development • Supplementary information

S2.6 Single-file simulations

To assess the role of plasmodesmatal fluxes within the individual tissue layers, we simulated auxin transport through a single file of cells, with PIN efflux carriers located on the downstream cell membranes. The model incorporates passive diffusion of protonated auxin across cell membranes, PIN-mediated transport of anionic auxin across cell membranes and plasmodesmatal diffusion of auxin between adjacent cell cytoplasms. Labelling the cells by $i = 1, \cdots, N$, we let $c_i(t)$ denote the auxin concentration in cell cytoplasm i and $f_i(t)$ denote the auxin concentration of the apoplast region neighbouring cell cytoplasms i and $i+1$, at time t. The auxin fluxes across each cell membrane are then given by

$$J_{cfi} =(B_1 P_{IAAH} + B_3 P_{PIN})c_i - (A_1 P_{IAAH} + A_3 P_{PIN}) f_i \quad \text{for} \quad i = 1, 2, \cdots, N-1, \tag{14}$$
$$J_{fci} =A_1 P_{IAAH} f_{i-1} - B_1 P_{IAAH} c_i \quad \text{for} \quad i = 2, 3, \cdots, N, \tag{15}$$

where J_{cfi} denotes the flux from cell i to apoplast region i and J_{fci} denotes the flux from apoplast region $i-1$ to cell i (see Band and King (2012) for the derivation of these flux terms). The auxin fluxes through plasmodesmata from cell i to cell $i+1$ are given by

$$J_i^{plas} = P_{plas} d(c_i - c_{i+1}) \quad \text{for} \quad i = 1, 2, \cdots, N-1, \tag{16}$$

where P_{plas} denotes the permeability per plasmodesmata and d denotes the density of plasmodesmata (which is taken to be a constant in this single-layer model).

The auxin dynamics are then governed by the following system of coupled ordinary differential equations (ODEs):

$$\frac{\mathrm{d}c_i}{\mathrm{d}t} = \frac{1}{l}(J_{fci} - J_{cfi} + J_{i-1}^{plas} - J_i^{plas}) \text{ for } i = 2, \cdots, N-1, \tag{17}$$
$$\frac{\mathrm{d}f_i}{\mathrm{d}t} = \frac{1}{\lambda}(J_{cfi} - J_{fc(i+1)}) \text{ for } i = 1, \cdots, N-1, \tag{18}$$

where l denotes the cell length and λ denotes the apoplast thickness.

These ODEs, (17), are simulated under the assumption that the concentration in cells $i = 1$ and $i = N$ are held fixed, $c_1(t) = 1$, $c_N(t) = 0$, and all other concentrations are initially zero, $c_i(0) = 0$ for $i = 2, 3, \cdots, N$, $f_i(0) = 0$ for $i = 1, 2, \cdots, N$. Figs. 3g, S12 show the simulation results for parameter values $N = 100$, $l = 20\,\mu$m, $\lambda = 0.5\,\mu m$, $d = 1\,\mu m^{-2}$ and the remaining parameters equal to those given in Table S2.

References

L R Band and J R King. Multiscale modelling of auxin transport in the plant-root elongation zone. *J Math Biol*, 65(4):743–85, Oct 2012.

Leah R Band, Darren M Wells, Antoine Larrieu, Jianyong Sun, Alistair M Middleton, Andrew P French, Géraldine Brunoud, Ethel Mendocilla Sato, Michael H Wilson, Benjamin Péret, Marina Oliva, Ranjan Swarup, Ilkka Sairanen, Geraint Parry, Karin Ljung, Tom Beeckman, Jonathan M Garibaldi, Mark Estelle, Markus R Owen, Kris Vissenberg, T Charlie Hodgman, Tony P Pridmore, John R King, Teva Vernoux, and Malcolm J Bennett. Root gravitropism is regulated by a transient lateral auxin gradient controlled by a tipping-point mechanism. *Proc Natl Acad Sci USA*, 109(12):4668–73, Mar 2012.

Development • Supplementary information

Leah R Band, Darren M Wells, John A Fozard, Teodor Ghetiu, Andrew P French, Michael P Pound, Michael H Wilson, Lei Yu, Wenda Li, Hussein I Hijazi, Jaesung Oh, Simon P Pearce, Miguel A Perez-Amador, Jeonga Yun, Eric Kramer, Jose M Alonso, Christophe Godin, Teva Vernoux, T Charlie Hodgman, Tony P Pridmore, Ranjan Swarup, John R King, and Malcolm J Bennett. Systems analysis of auxin transport in the Arabidopsis root apex. *Plant Cell*, 26(3):862–75, Mar 2014.

Géraldine Brunoud, Darren M Wells, Marina Oliva, Antoine Larrieu, Vincent Mirabet, Amy H Burrow, Tom Beeckman, Stefan Kepinski, Jan Traas, Malcolm J Bennett, and Teva Vernoux. A novel sensor to map auxin response and distribution at high spatio-temporal resolution. *Nature*, 482(7383):103–6, Jan 2012.

Rosemary J Dyson, Gema Vizcay-Barrena, Leah R Band, Anwesha N Fernandes, Andrew P French, John A Fozard, T Charlie Hodgman, Kim Kenobi, Tony P Pridmore, Michael Stout, Darren M Wells, Michael H Wilson, Malcolm J Bennett, and Oliver E Jensen. Mechanical modelling quantifies the functional importance of outer tissue layers during root elongation and bending. *New Phytol*, 202(4):1212–22, Jun 2014.

Eric M Kramer, Nicholas L Frazer, and Tobias I Baskin. Measurement of diffusion within the cell wall in living roots of arabidopsis thaliana. *J Exp Bot*, 58(11):3005–15, 2007.

Nathan Mellor, Leah R Band, Aleš Pěnčík, Ondřej Novák, Afaf Rashed, Tara Holman, Michael H Wilson, Ute Voß, Anthony Bishopp, John R King, Karin Ljung, Malcolm J Bennett, and Markus R Owen. Dynamic regulation of auxin oxidase and conjugating enzymes atDAO1 and GH3 modulates auxin homeostasis. *Proc Natl Acad Sci U S A*, 113(39):11022–7, 09 2016.

NA Omelyanchuk, VV Kovrizhnykh, EA Oshchepkova, T Pasternak, K Palme, and VV Mironova. A detailed expression map of the pin1 auxin transporter in arabidopsis thaliana root. *BMC Plant Biology*, (Suppl 1):5, 2016.

Michael P Pound, Andrew P French, Darren M Wells, Malcolm J Bennett, and Tony P Pridmore. CellSeT: novel software to extract and analyze structured networks of plant cells from confocal images. *Plant Cell*, 24(4):1353–61, Apr 2012.

Christophe Pradal, Samuel Dufour-Kowalski, Frédéric Boudon, Christian Fournier, and Christophe Godin. OpenAlea: a visual programming and component-based software platform for plant modelling. *Functional Plant Biology*, 35(10):751, 2008. ISSN 1445-4408.

Heidi L Rutschow, Tobias I Baskin, and Eric M Kramer. Regulation of solute flux through plasmodesmata in the root meristem. *Plant Physiol*, 155(4):1817–26, Apr 2011.

Anna N Stepanova, Joyce Robertson-Hoyt, Jeonga Yun, Larissa M Benavente, De-Yu Xie, Karel Dolezal, Alexandra Schlereth, Gerd Jürgens, and Jose M Alonso. TAA1-mediated auxin biosynthesis is essential for hormone crosstalk and plant development. *Cell*, 133 (1):177–91, Apr 2008.

Ranjan Swarup, Eric M Kramer, Paula Perry, Kirsten Knox, H M Ottoline Leyser, Jim Haseloff, Gerrit T S Beemster, Rishikesh Bhalerao, and Malcolm J Bennett. Root gravitropism requires lateral root cap and epidermal cells for transport and response to a mobile auxin signal. *Nat Cell Biol*, 7(11):1057–65, Nov 2005.

Wei Xuan, Leah R Band, Robert P Kumpf, Daniël Van Damme, Boris Parizot, Gieljan De Rop, Davy Opdenacker, Barbara K Möller, Noemi Skorzinski, Maria F Njo, Bert De Rybel, Dominique Audenaert, Moritz K Nowack, Steffen Vanneste, and Tom Beeckman. Cyclic programmed cell death stimulates hormone signaling and root development in Arabidopsis. *Science*, 351(6271):384–7, Jan 2016.

T. Zhu, W. J. Lucas, and T. L. Rost. Directional cell-to-cell communication in the Arabidopsis root apical meristem i. an ultrastructural and functional analysis. *Protoplasma*, 203(1):35–47, Mar 1998. ISSN 1615-6102.

Development • Supplementary information

Supplementary Figures

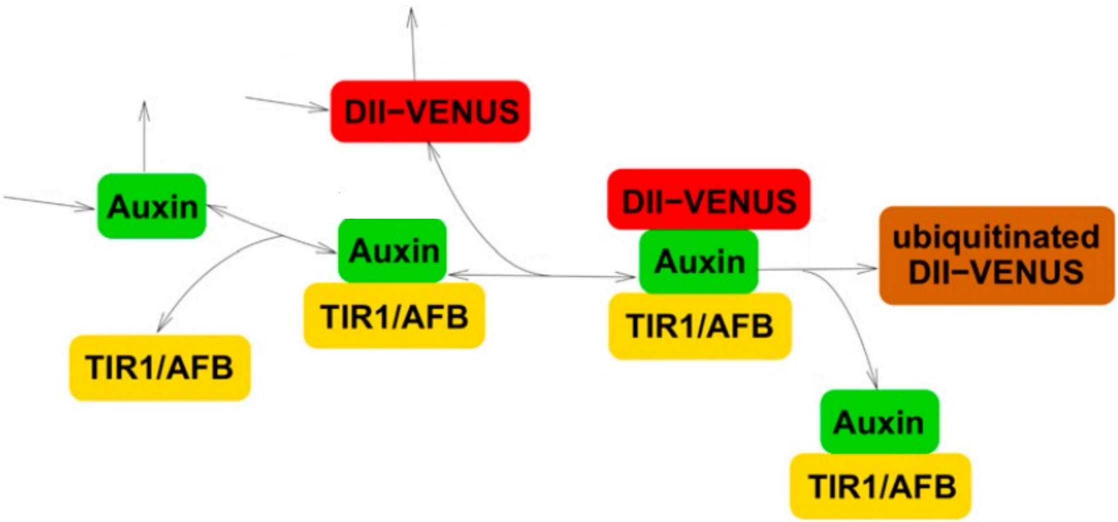

Figure S1: Network of interactions through which auxin degrades DII-VENUS.

Development • Supplementary information

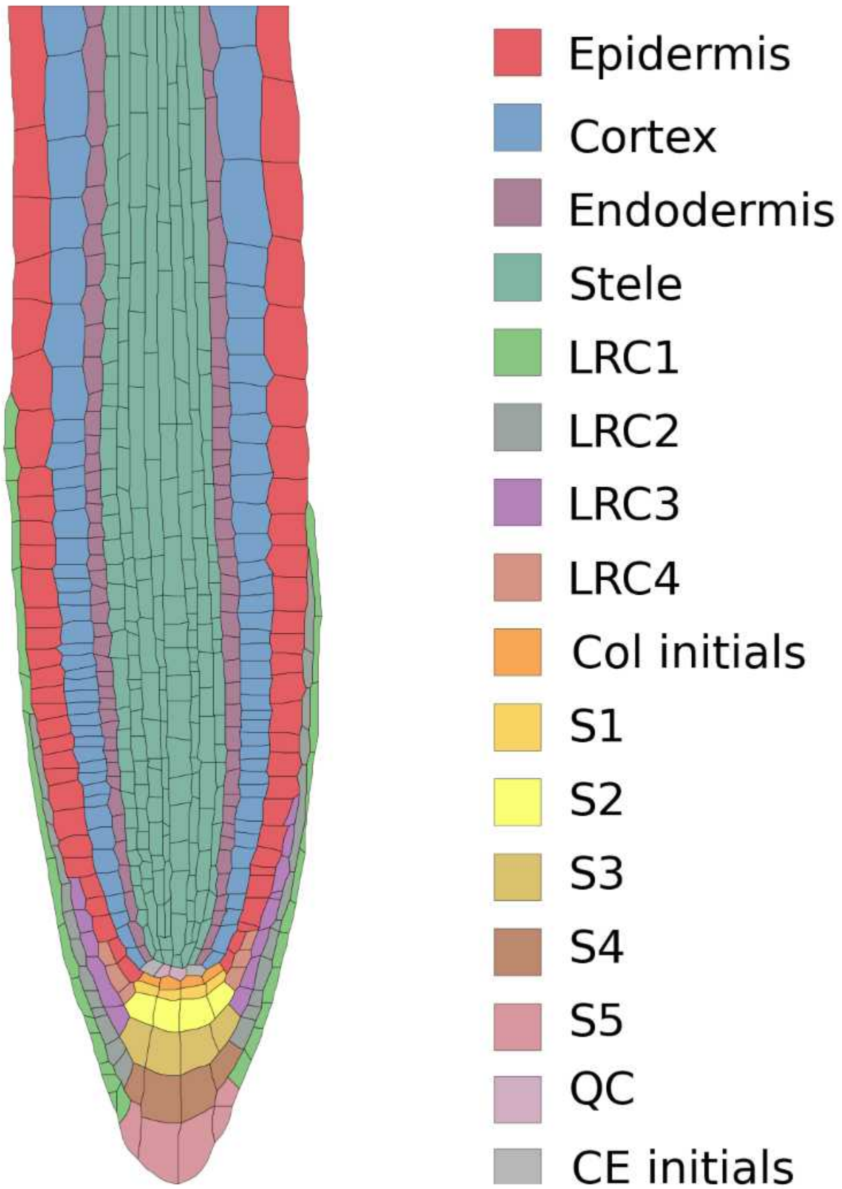

Figure S2: Schematic diagram showing cell types within the *Arabidopsis* root tip used in the model.

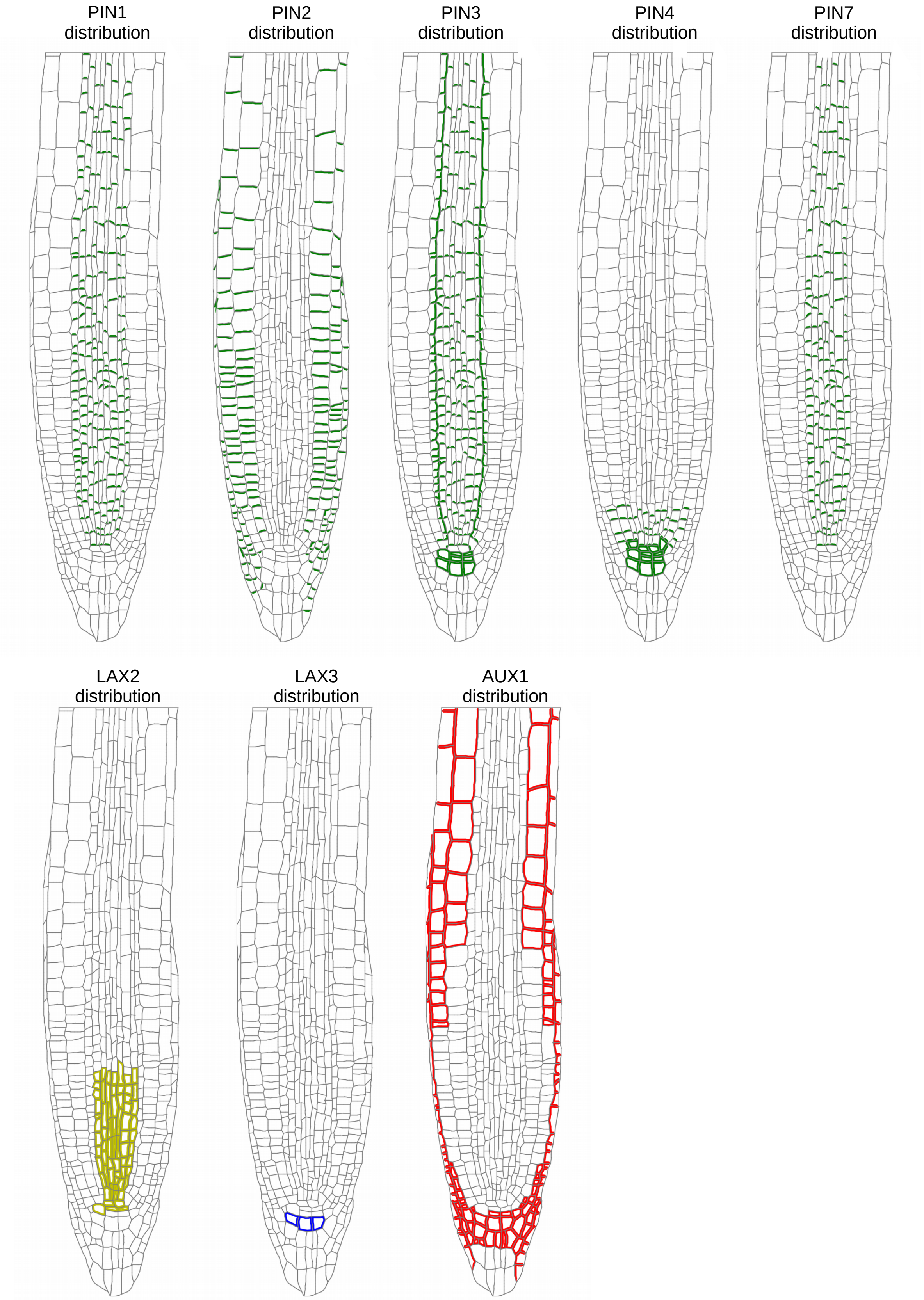

Figure S3: Root templates showing individual PIN, AUX1 and LAX distributions.

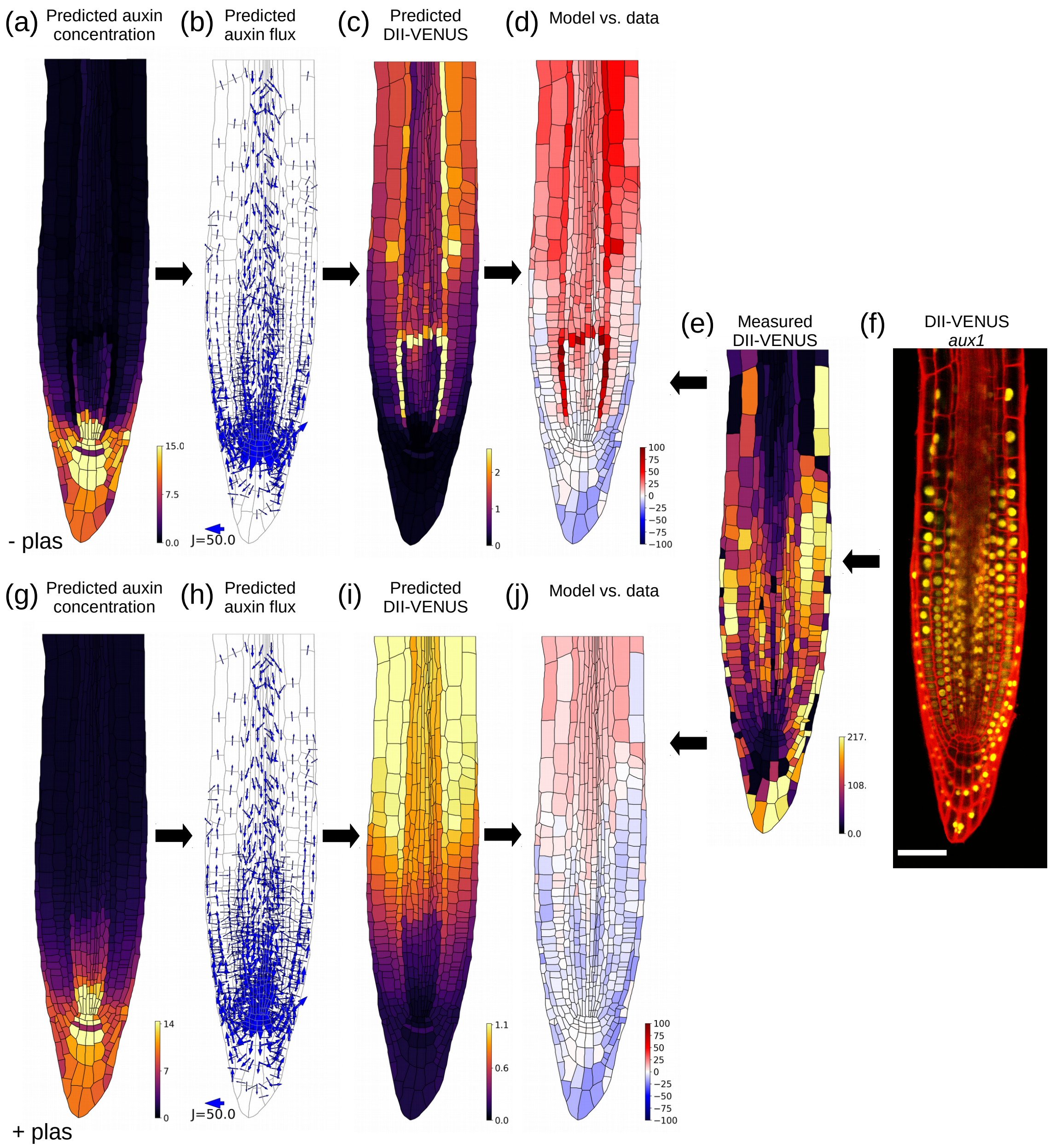

Figure S4: Predicted auxin and DII-VENUS distributions in the *aux1* mutant without (a-d) and with (g-j) plasmodesmata. (a,g). Predicted steady-state auxin distribution (b,h). Predicted auxin fluxes (c,i). Predicted DII-VENUS distribution (d,j). Difference between predicted and observed DII-VENUS distribution (from predictions in panels c,i and data in panel (e) (e). Quantification of DII-VENUS distribution using image in f (quantified using CellSet image segmentation software (Pound et al 2012). (f). DII-VENUS confocal image. Scale bar 50 μm.

Development • Supplementary information

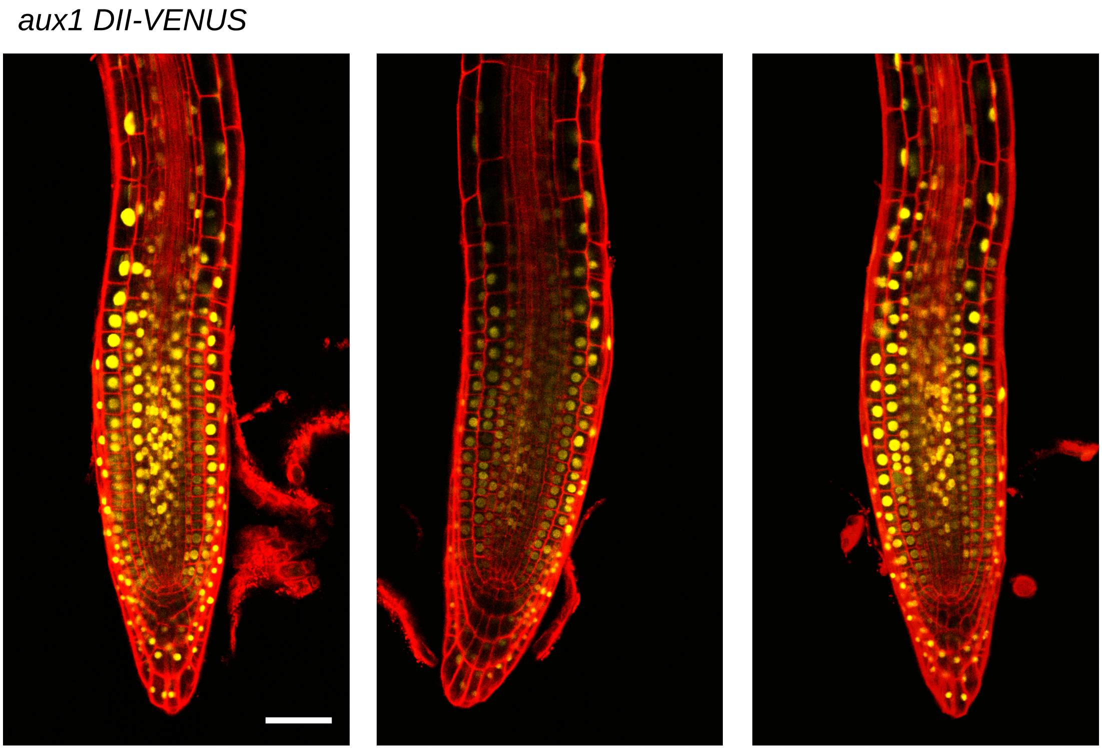

Figure S5: Replicates of the DII-VENUS distribution in *aux1*. Scale bar 50 μm.

Development • Supplementary information

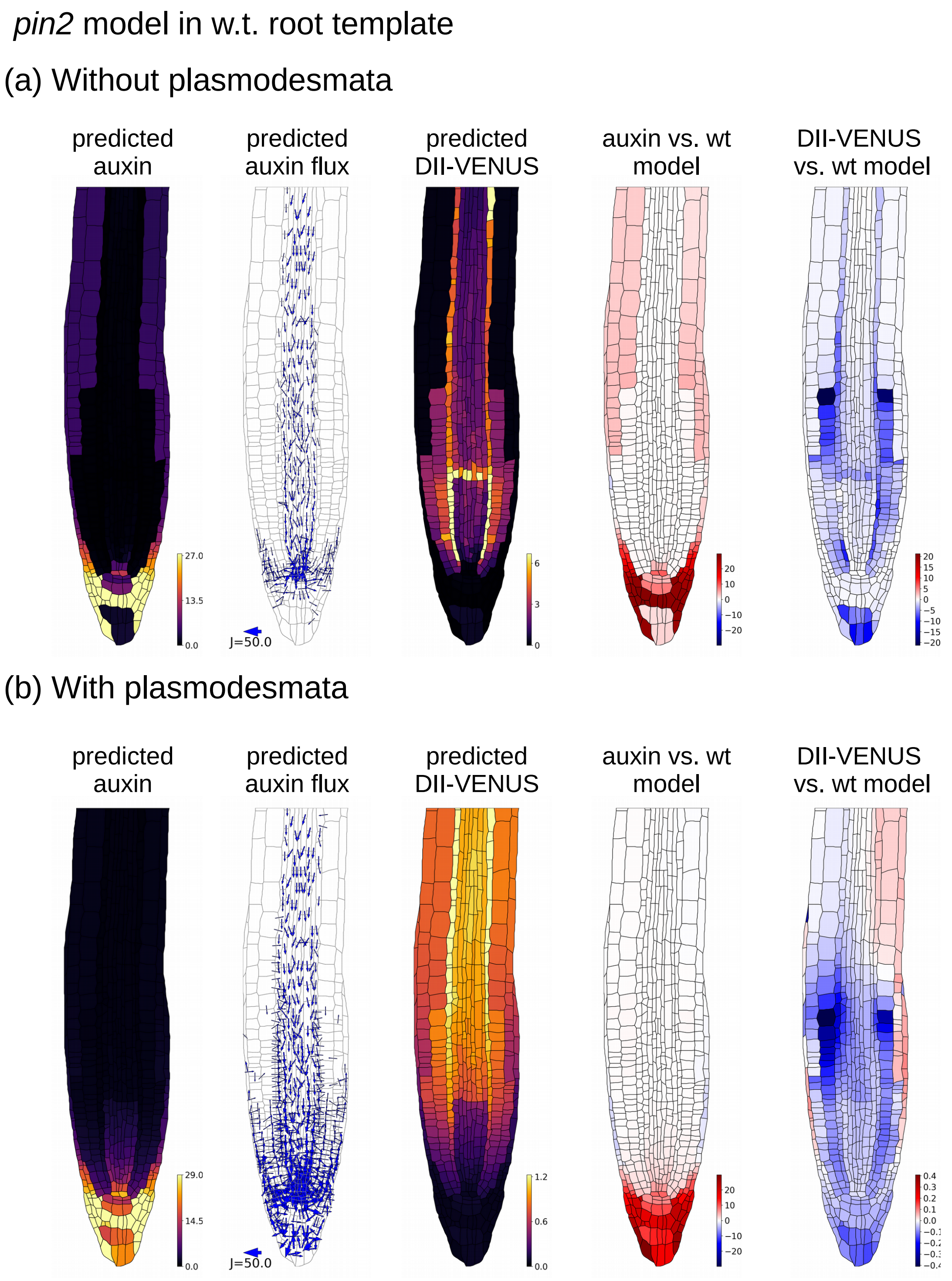

Figure S6: Predicted auxin concentrations, auxin fluxes, DII- VENUS, auxin concentrations relative to wild type model, and DII-VENUS concentrations relative to wild type model using the *pin2* model in the wild type root template. (a) shows the model without plasmodesmata, (b) shows the model with plasmodesmata.

Development • Supplementary information

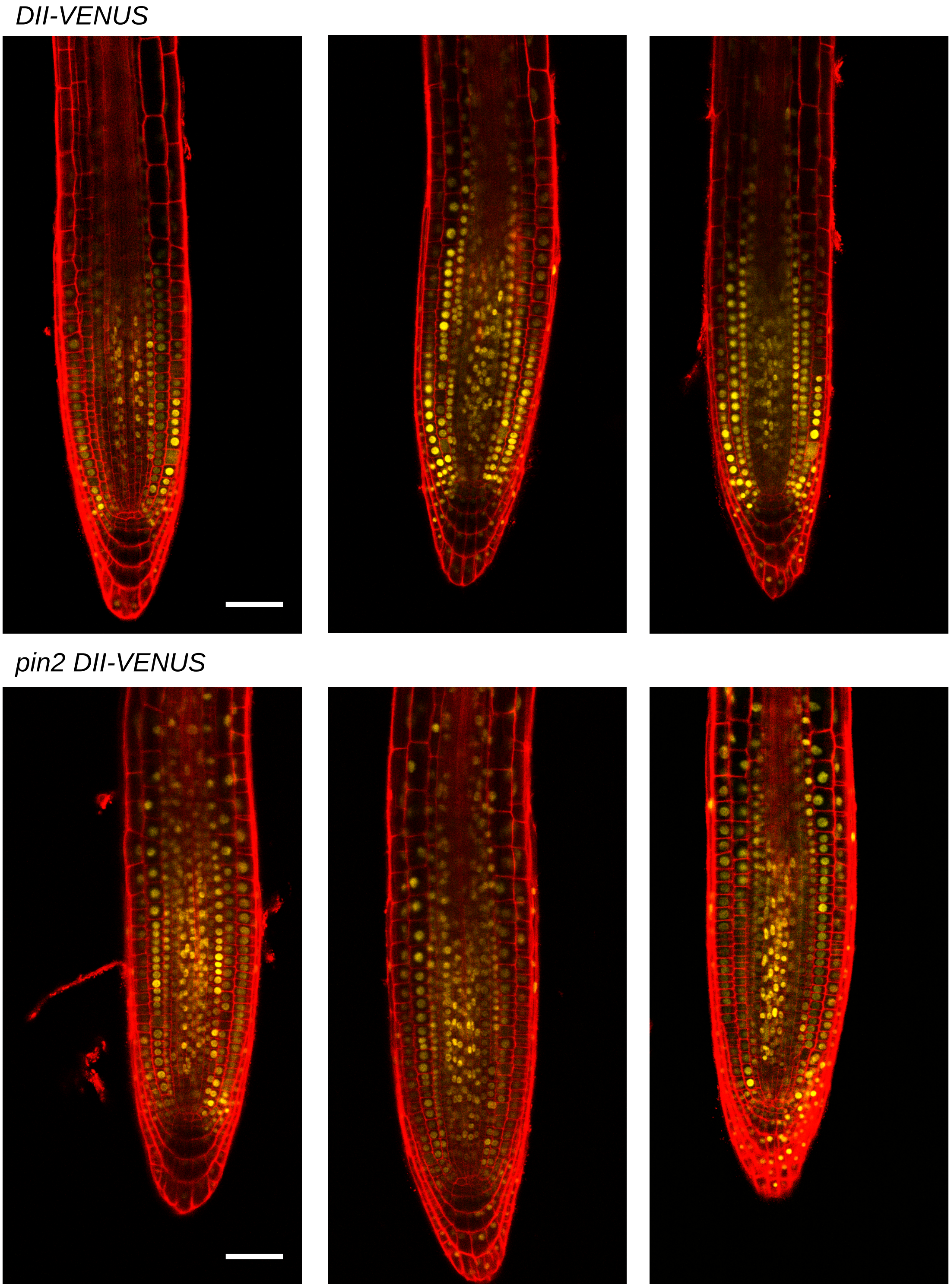

Figure S7: Replicates of the DII-VENUS distribution in wild type and *pin2*. Scale bars 50 μm.

Development • Supplementary information

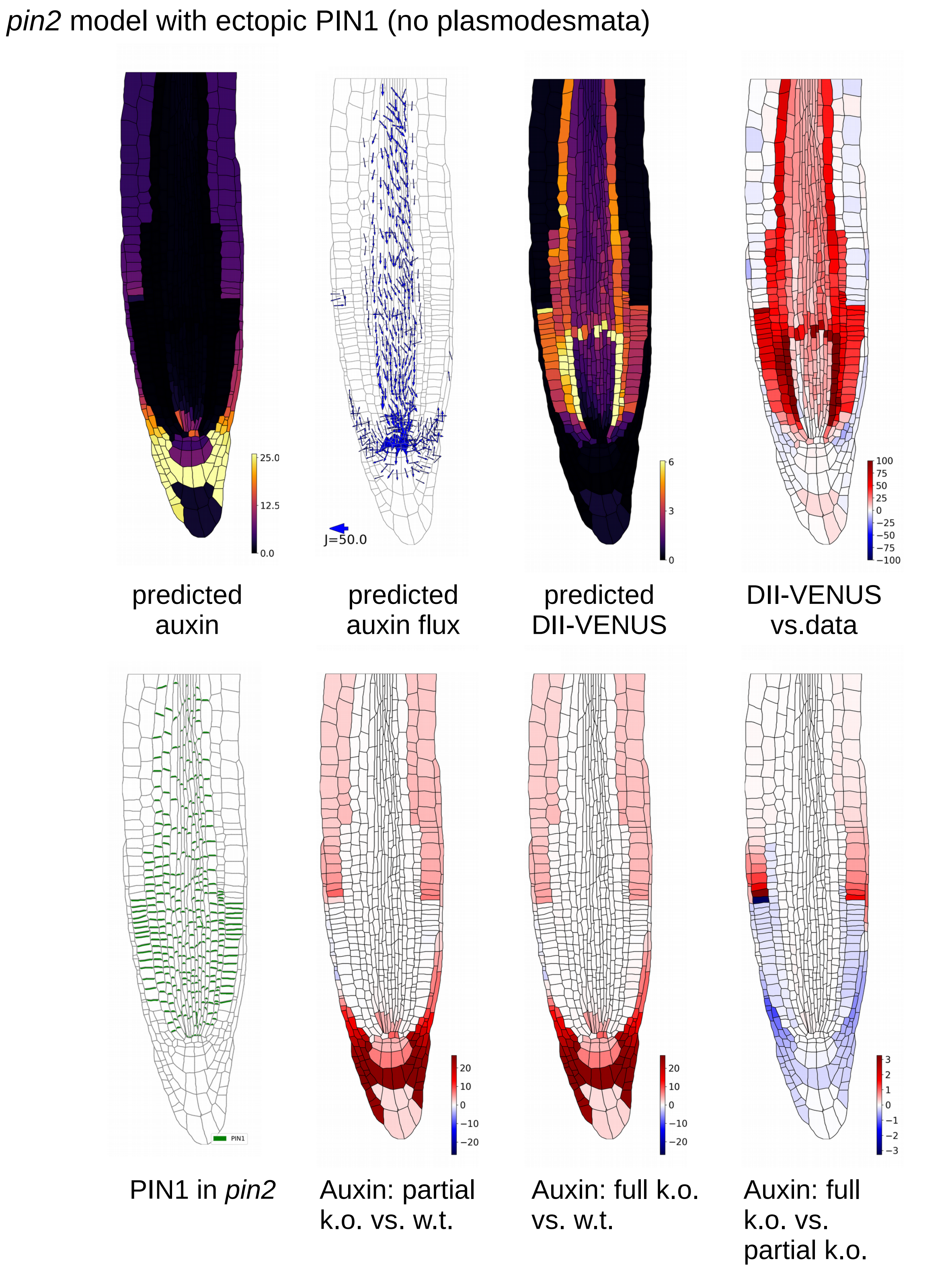

Figure S8: Model results without plasmodemata with the ectopically expressed PIN1 spatial distribution previously observed in *pin2*. Lower panels show (left to right) the PIN1 in *pin2* distribution, auxin in the model with ectopic PIN1 (labelled 'partial k.o.') relative to wild type, the predicted auxin in the model without ectopic PIN1 (labelled 'full k.o.') relative to wild type, and the predicted auxin in the full k.o. model relative to the partial k.o. model.

Development • Supplementary information

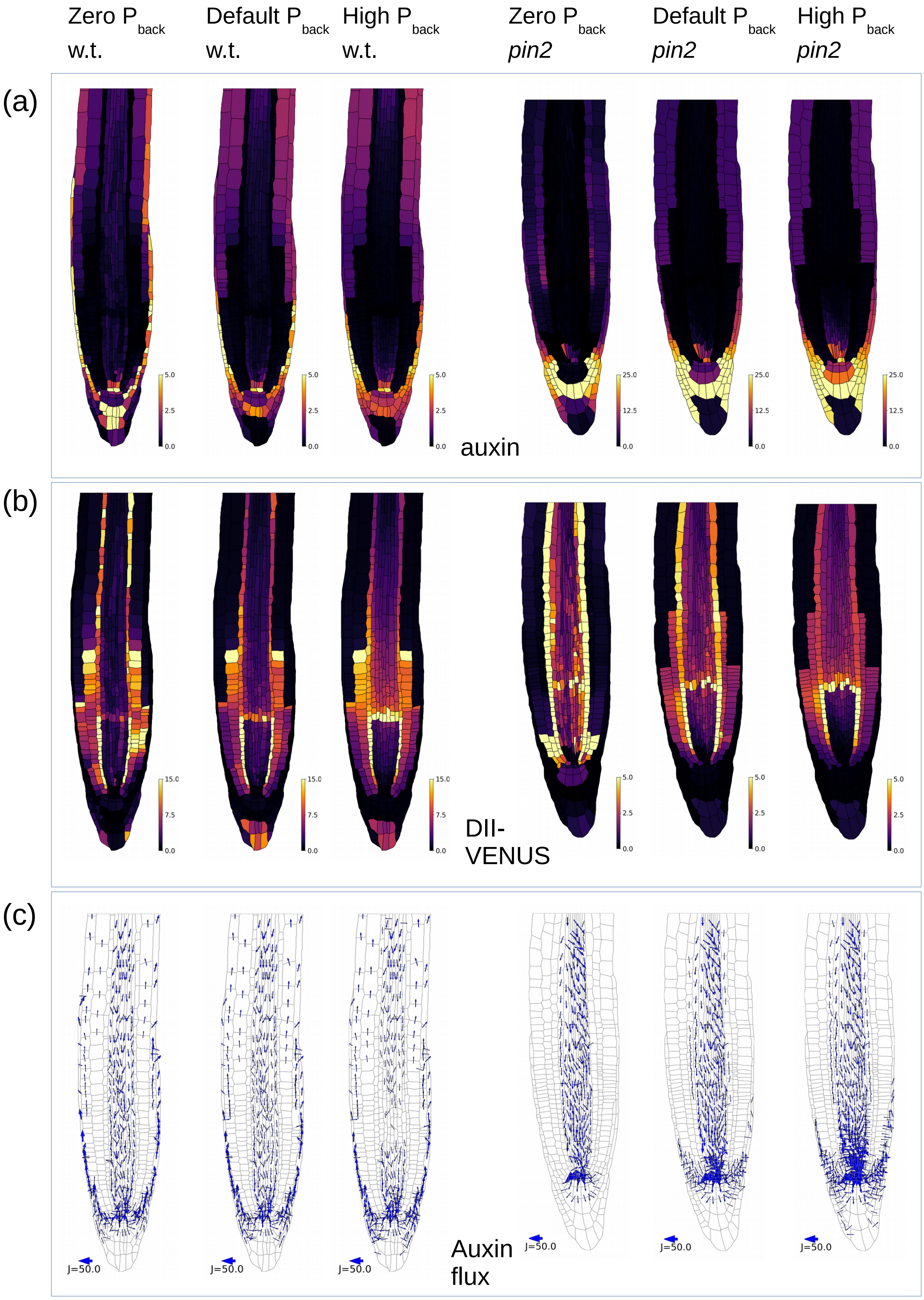

Figure S9: Effect of P_{back} in wild type and *pin2* models without plasmodesmata on predicted auxin (a), DII-VENUS (b) and auxin flux (c). Default P_{back} is 30% the value of PIN permeability (0.168 μms^{-1}), while high P_{back} is set equal to the PIN permeability (0.56 μms^{-1})

Development • Supplementary information

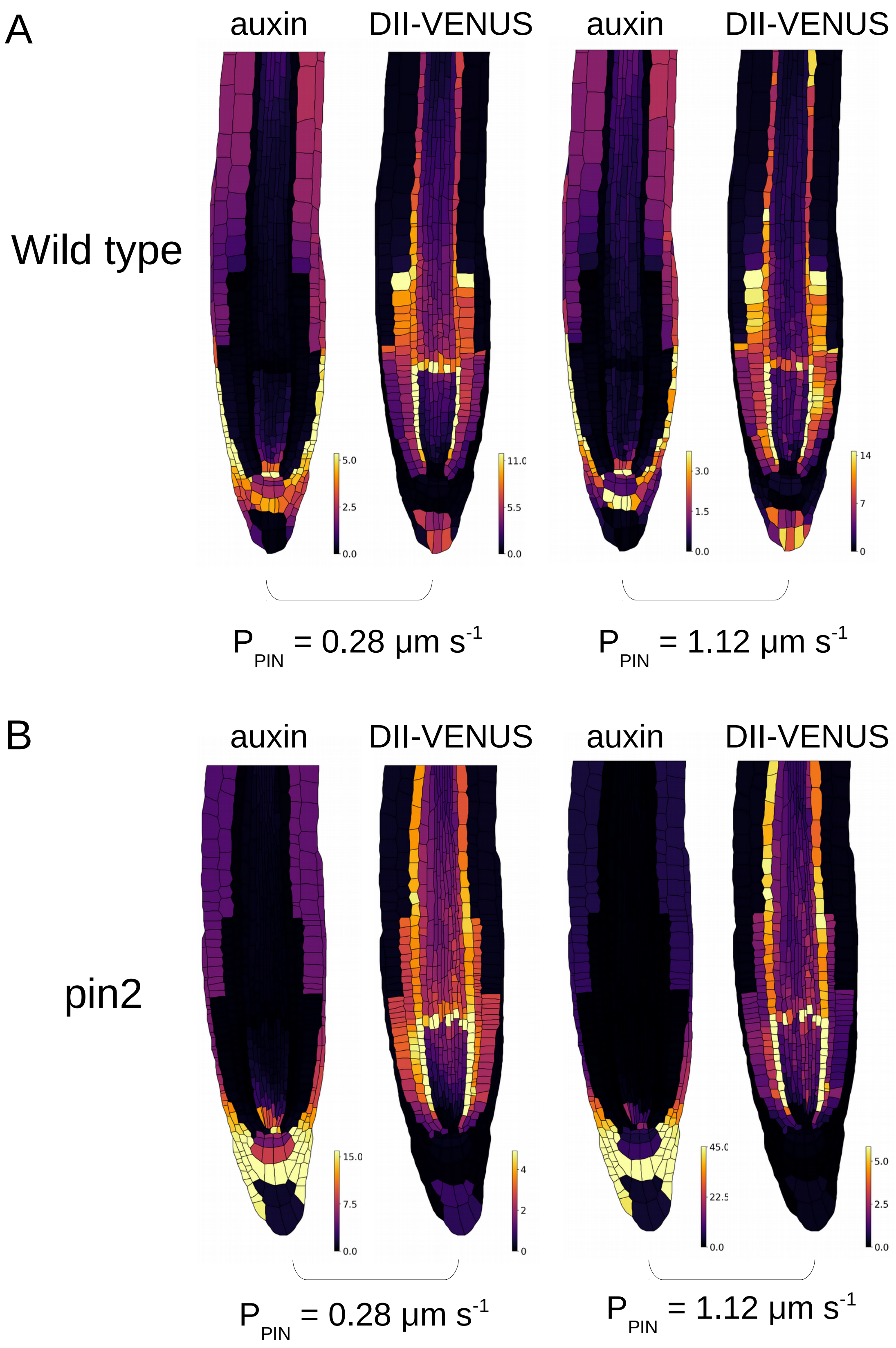

Figure S10: Predicted auxin and DII-VENUS in (A) wild type, and (B) *pin2*, using the model without plasmodesmata, with values of the PIN permeability (P_{PIN}) set to half (left) and double (right) the estimated value of 0.56 $\mu m s^{-1}$. In each case the remaining model parameters are as given in Table 2, Supplementary Modelling information.

Development • Supplementary information

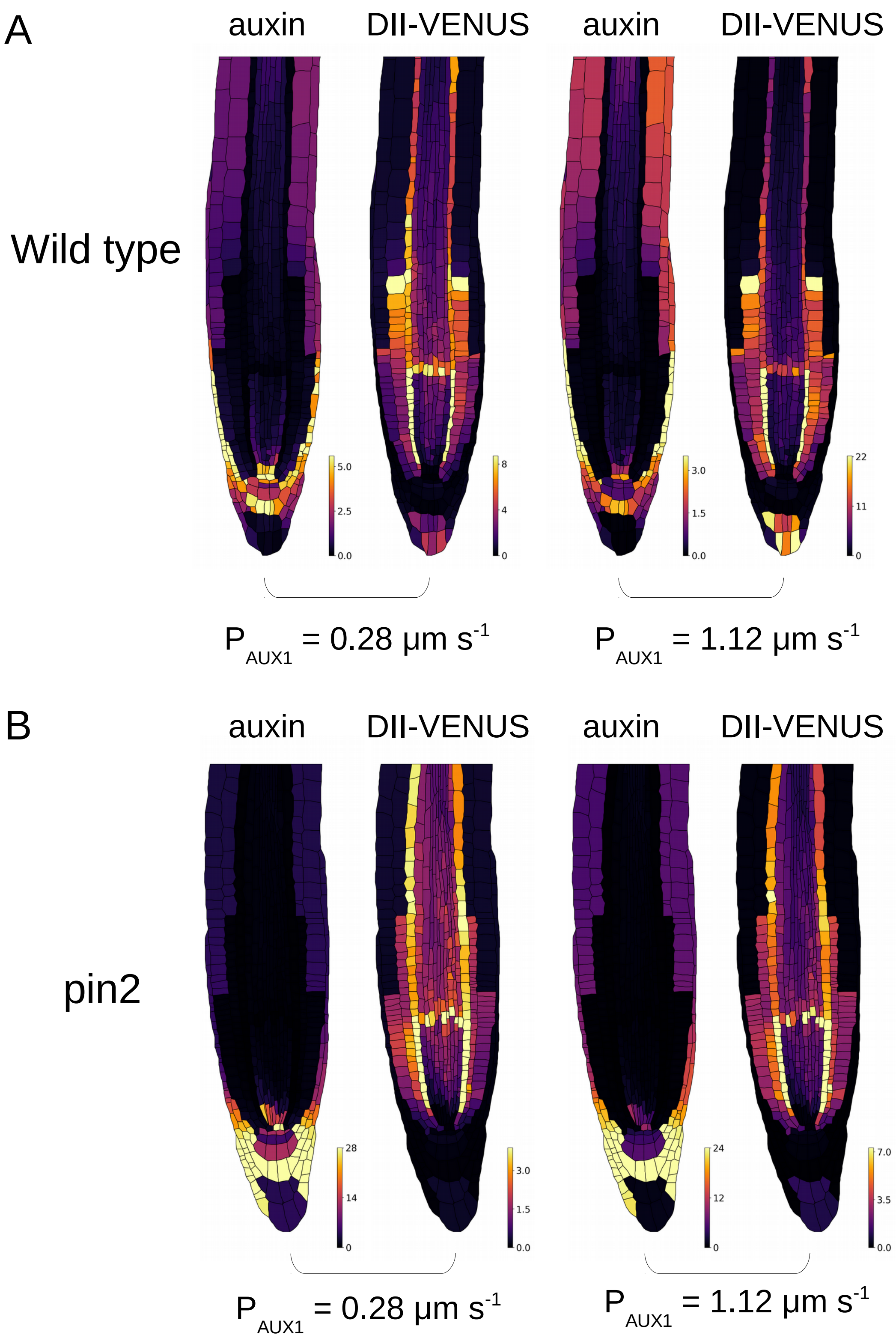

Figure S11: Predicted auxin and DII-VENUS in (A) wild type, and (B) *pin2*, using the model without plasmodesmata, with values of the PIN permeability (P_{AUX1}) set to half (left) and double (right) the estimated value of 0.56 $\mu m s^{-1}$. In each case the remaining model parameters are as given in Table 2, Supplementary Modelling information.

Development • Supplementary information

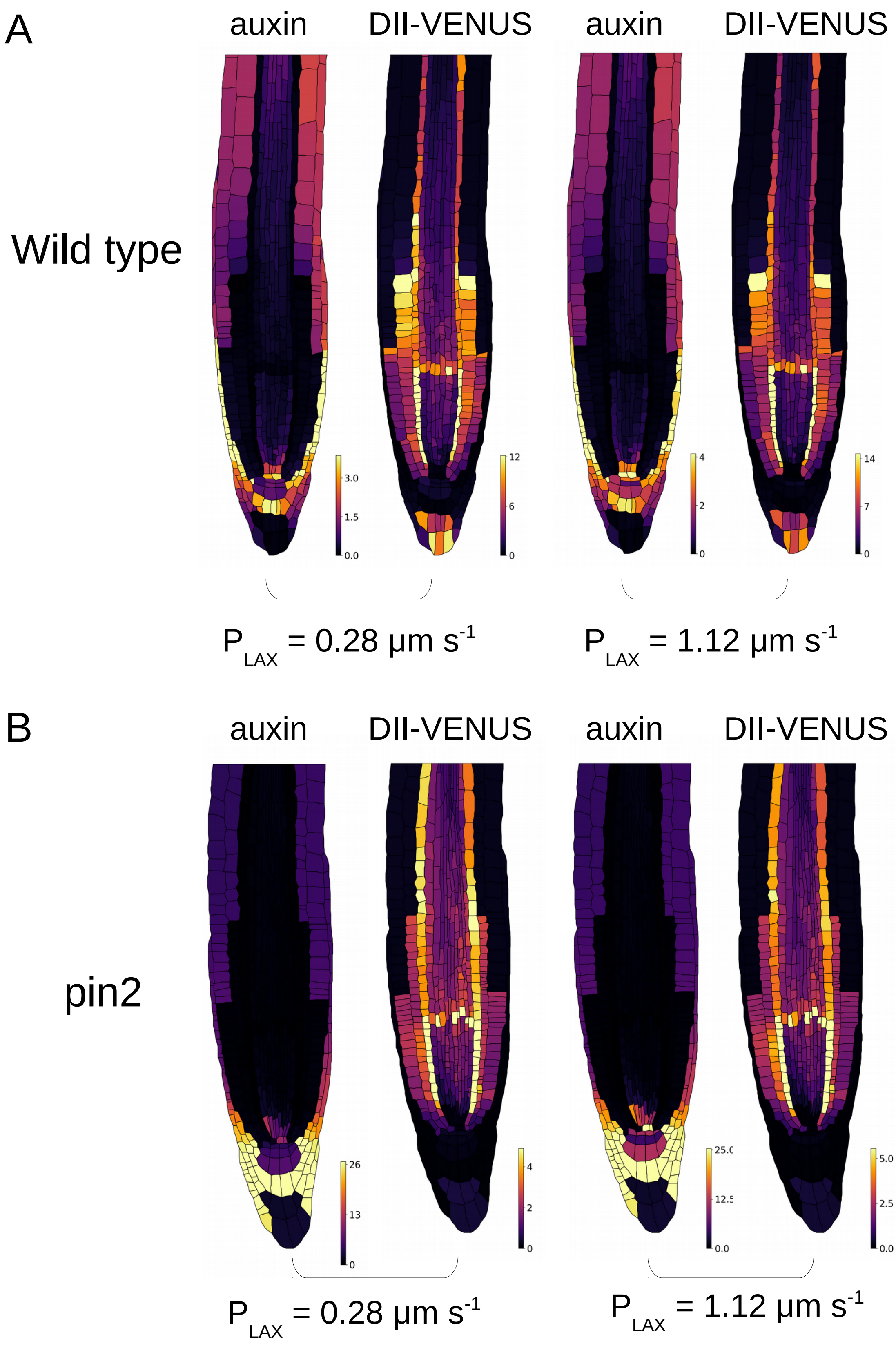

Figure S12: Predicted auxin and DII-VENUS in (A) wild type, and (B) *pin2*, using the model without plasmodesmata, with values of the PIN permeability (P_{LAX}) set to half (left) and double (right) the estimated value of 0.56 $\mu m s^{-1}$. In each case the remaining model parameters are as given in Table 2, Supplementary Modelling information.

Development • Supplementary information

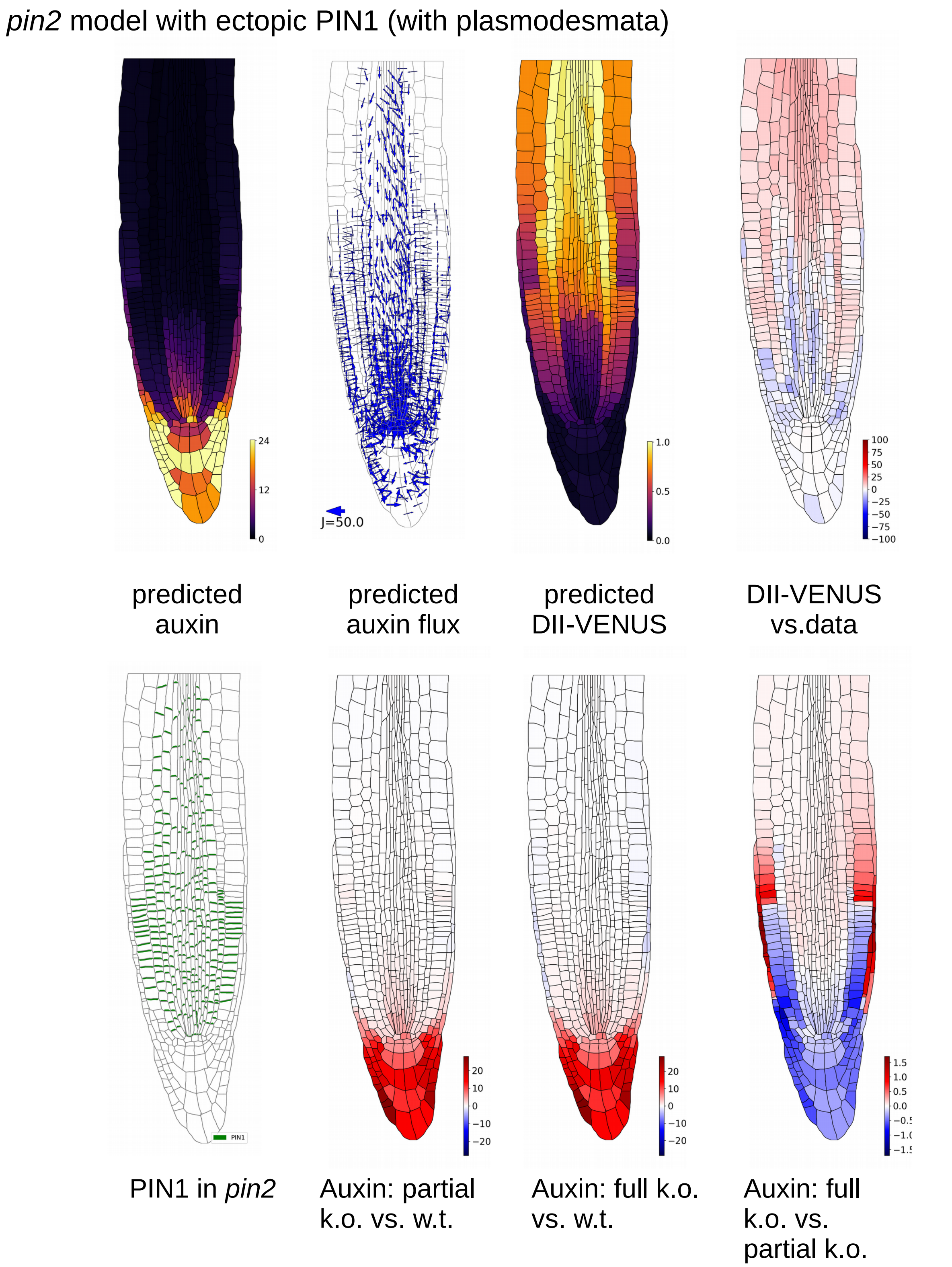

Figure S13: Model results with plasmodemata with the ectopically expressed PIN1 spatial distribution previously observed in *pin2*. Lower panels show (left to right) the PIN1 in *pin2* distribution, auxin in the model with ectopic PIN1 (labelled 'partial k.o.') relative to wild type, the predicted auxin in the model without ectopic PIN1 (labelled 'full k.o.') relative to wild type, and the predicted auxin in the full k.o. model relative to the partial k.o. model.

Development • Supplementary information

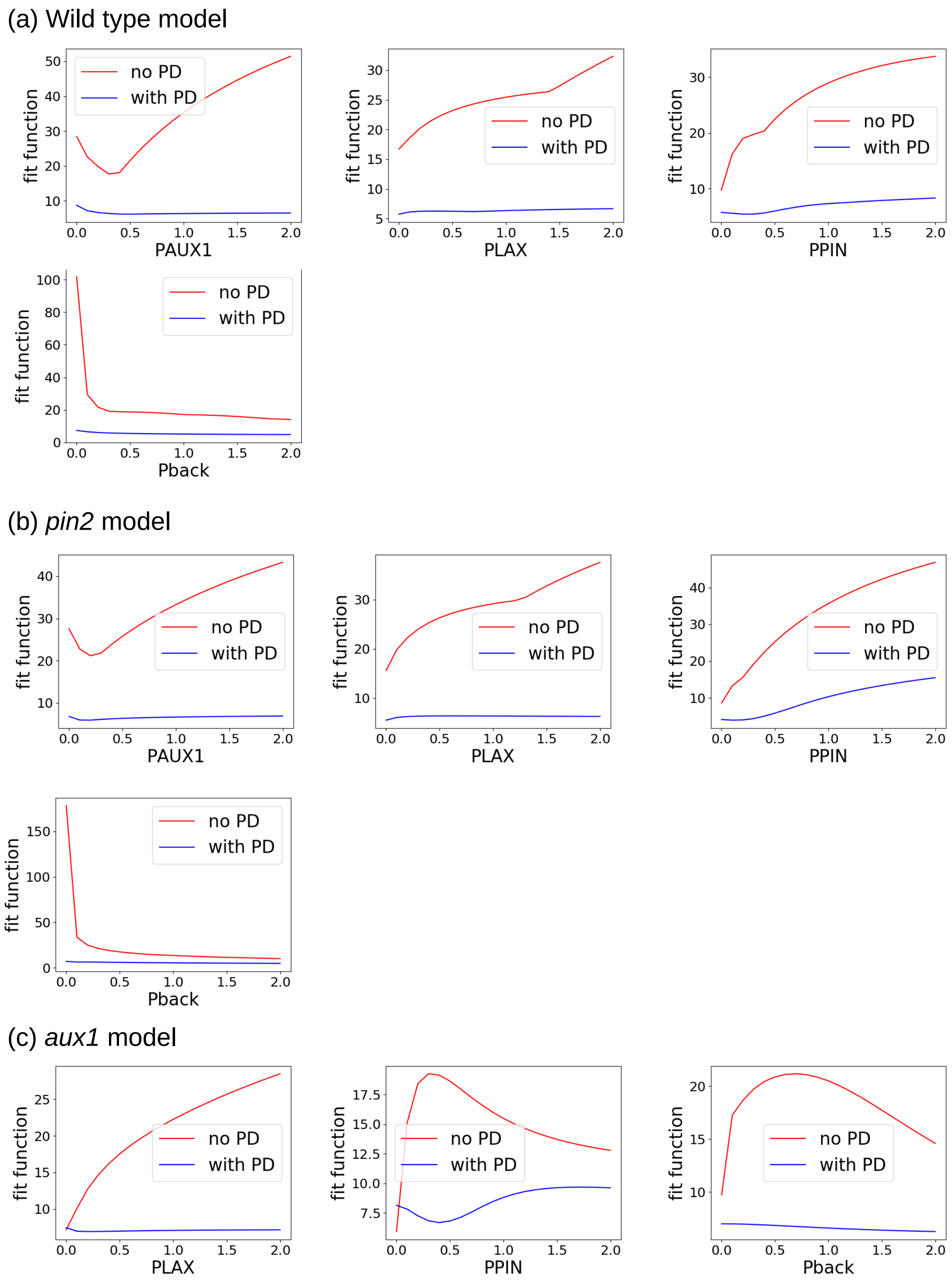

Figure S14: Evaluation of fit function (See main text, SI for details) in (a) wild type, (b) *pin2* and (c) *aux1* models with and without plasmodesmata (PD), for a range of values of the model permeability parameters P_{AUX1}, P_{LAX}, P_{PIN} and P_{back}. P_{AUX1} omitted for the *aux1* model as it is redundant in that case. In each case the remaining model parameters are as given in Table 2, Supplementary Modelling information.

Development • Supplementary information

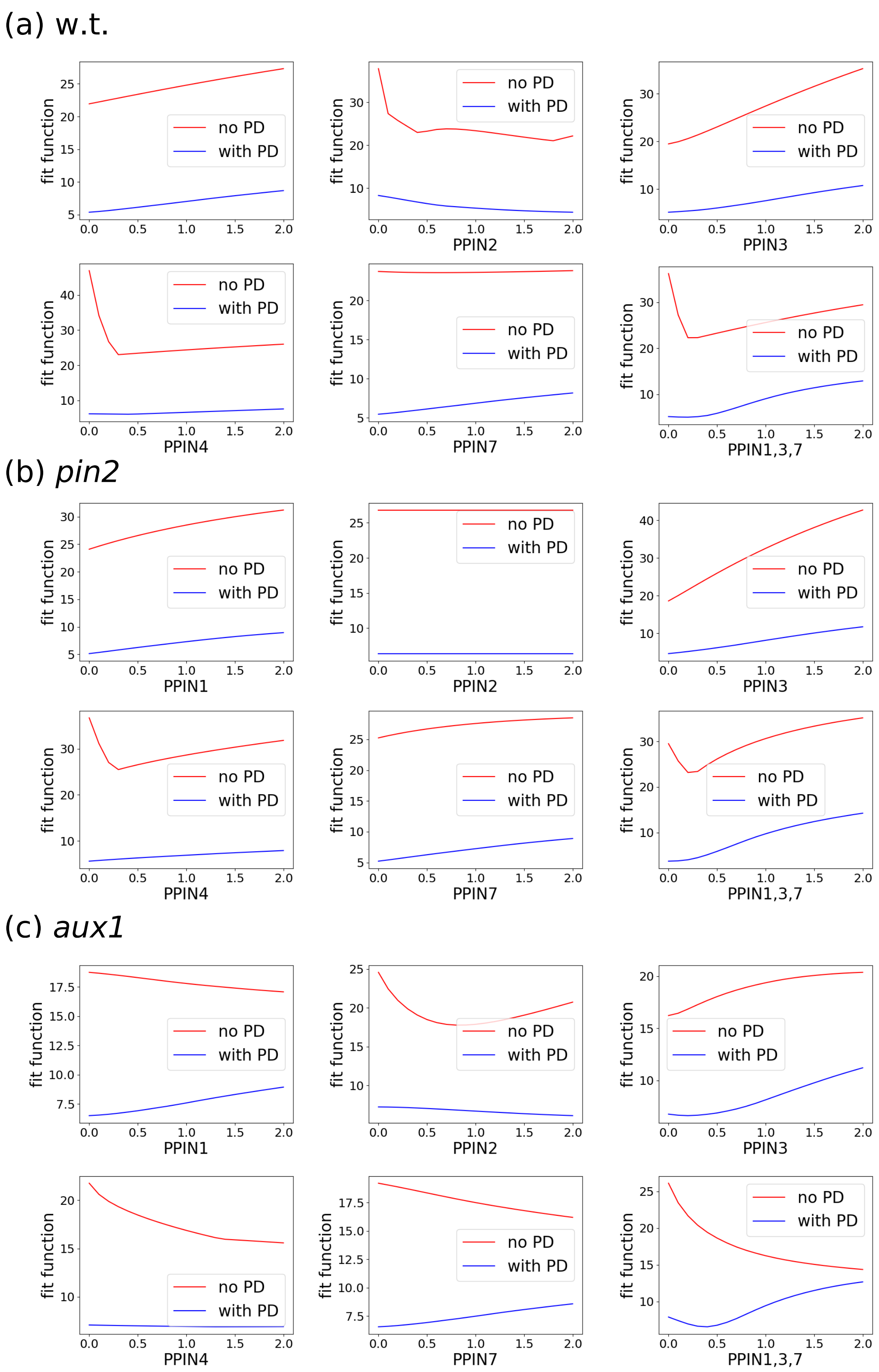

Figure S15: Evaluation of fit function (See main text, SI for details) in (a) wild type, (b) *pin2* and (c) *aux1* models with and without plasmodesmata (PD), for a range of values of the individual PIN permeability parameters P_{PIN1}, P_{PIN2}, P_{PIN3}, P_{PIN4}, P_{PIN7} and all PINs in the stele combined (P_{PIN1}, P_{PIN2} and P_{PIN7}). In each case the other PIN permeabilites are equal to P_{PIN} as given in Table 2, Supplementary Modelling information.

Development • Supplementary information

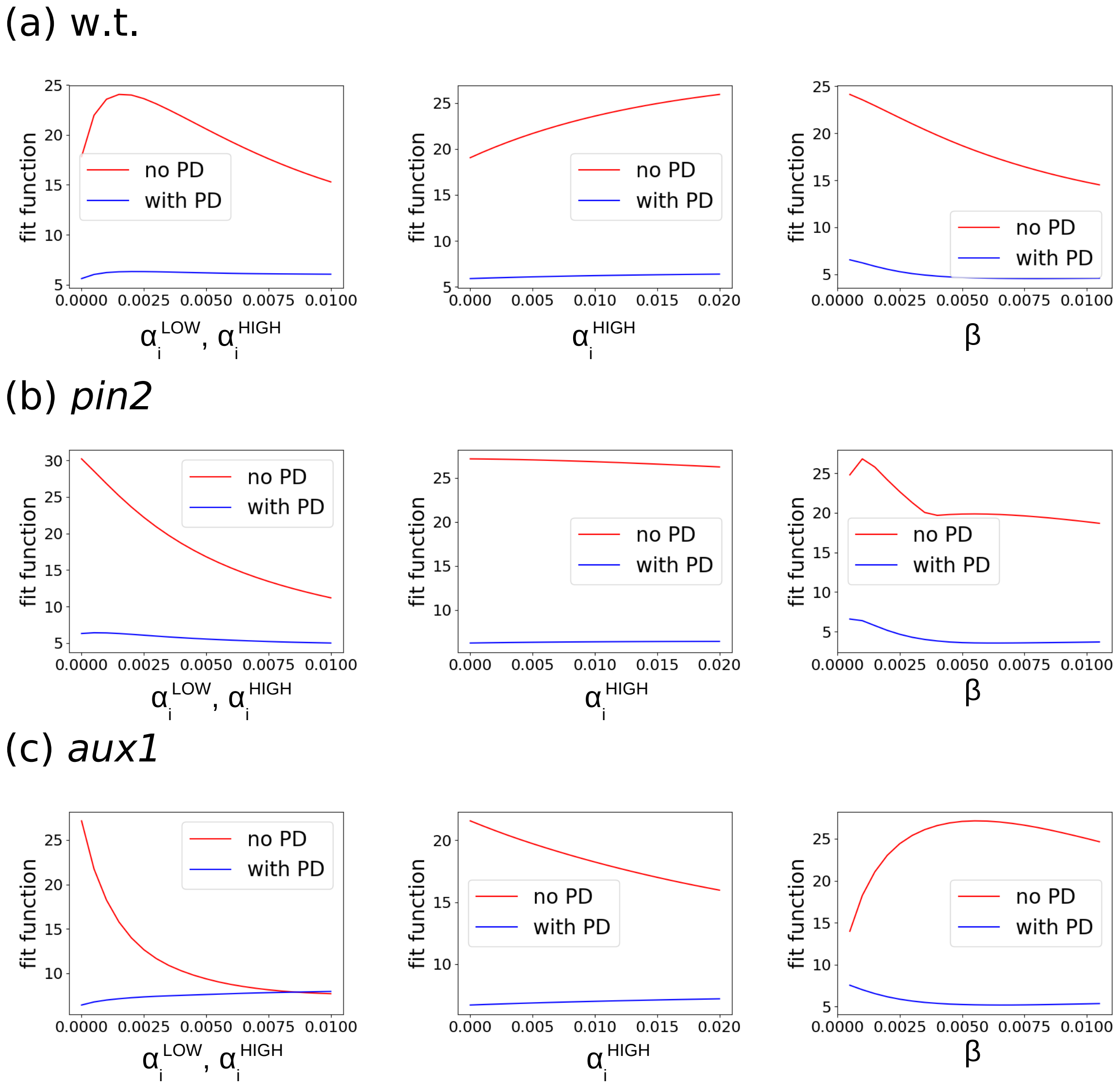

Figure S16: Evaluation of fit function (See main text, SI for details) in (a) wild type, (b) *pin2* and (c) *aux1* models with and without plasmodesmata (PD), for a range of values of the parameters α_i^{LOW} (auxin production outside QC, initials and outer LRC) in conjuction with α_i^{HIGH} (auxin production in QC, initials and outer LRC), α_i^{HIGH} alone, and β (auxin degradation). In the plots where α_i^{LOW} and α_i^{HIGH} are varied in conjuction, α_i^{HIGH} is always tenfold the stated value of α_i^{LOW}. In each case the remaining model parameters are as given in Table 2, Supplementary Modelling information.

Development • Supplementary information

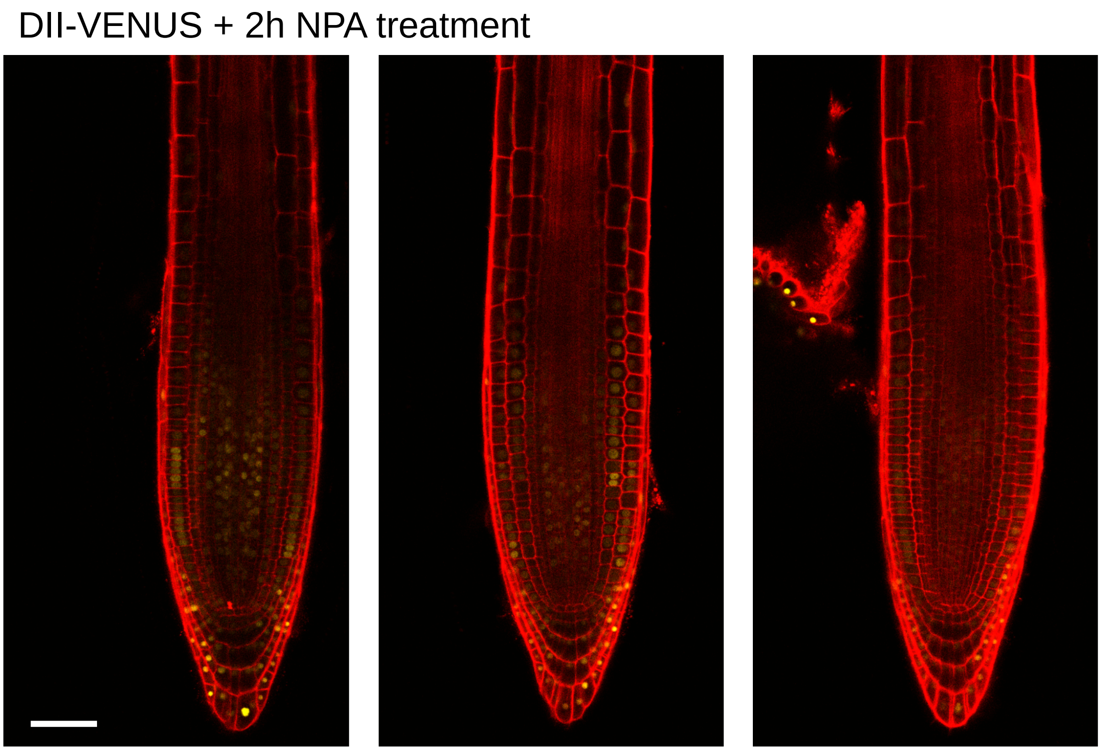

Figure S17: Replicate images of DII-VENUS roots following 2 hour treatment with 2 μM NPA. Scale bar 50 μm.

Development • Supplementary information

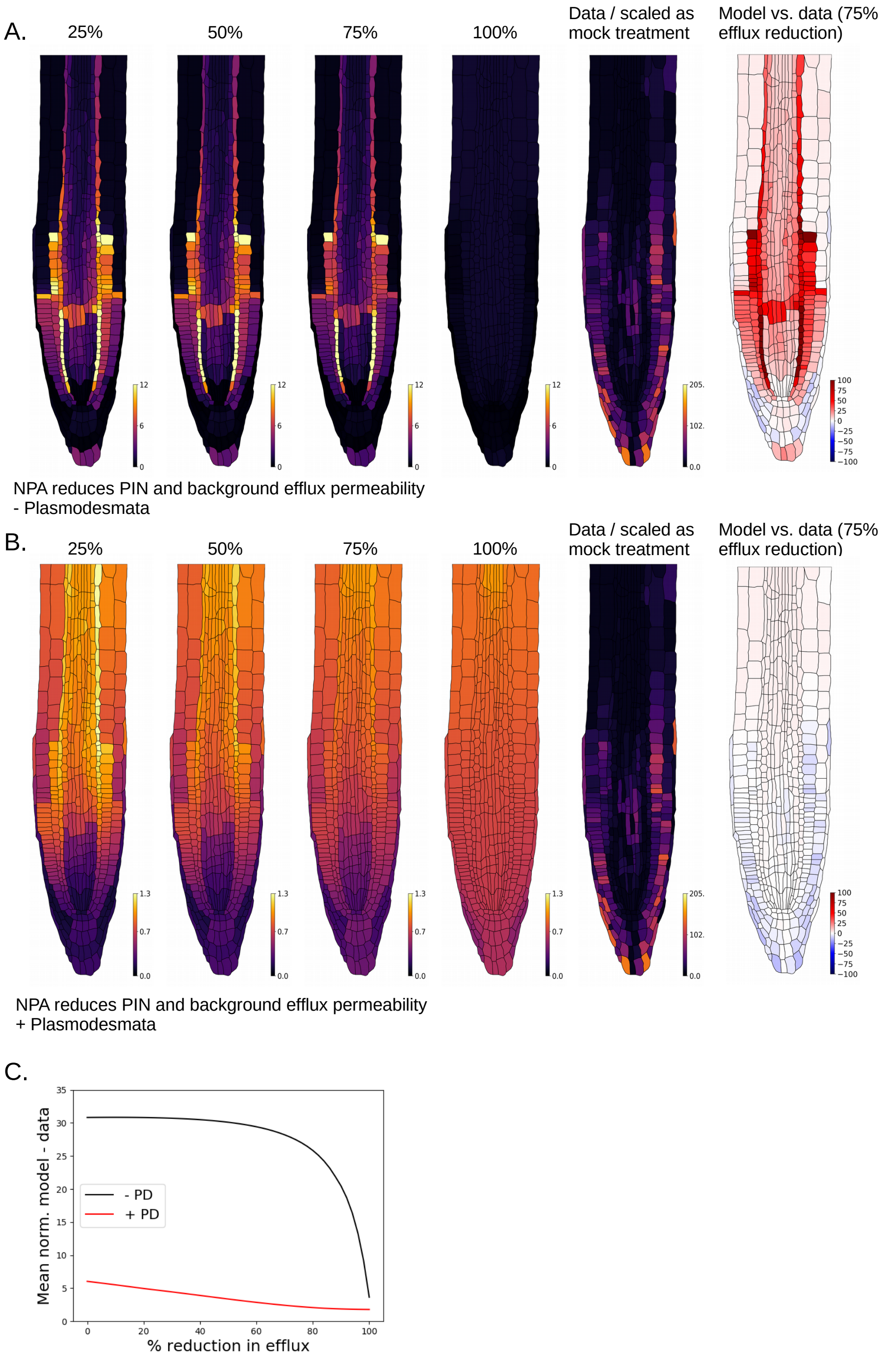

Figure S18: Simulated NPA treatment at four levels of efficacy in blocking PIN and background carrier efflux (25%, 50%, 75%, 100%, where %100 represents a complete block in efflux activity), in a model without plasmodesmata (A), and with plasmodesmata (B). The experimentally measured DII-VENUS following 2-hour treatment with 2 μM NPA is shown (on the same scale as the mock treated roots in Figure 1F by way of comparison), along with a normalised cell-by-cell comparison of the data versus the model with an estimated 75% reduction in carrier-mediated efflux. (C). Quantification of model fit versus percentage reduction in efflux carrier efficiency for models with and without plasmodesmata.

Development • Supplementary information

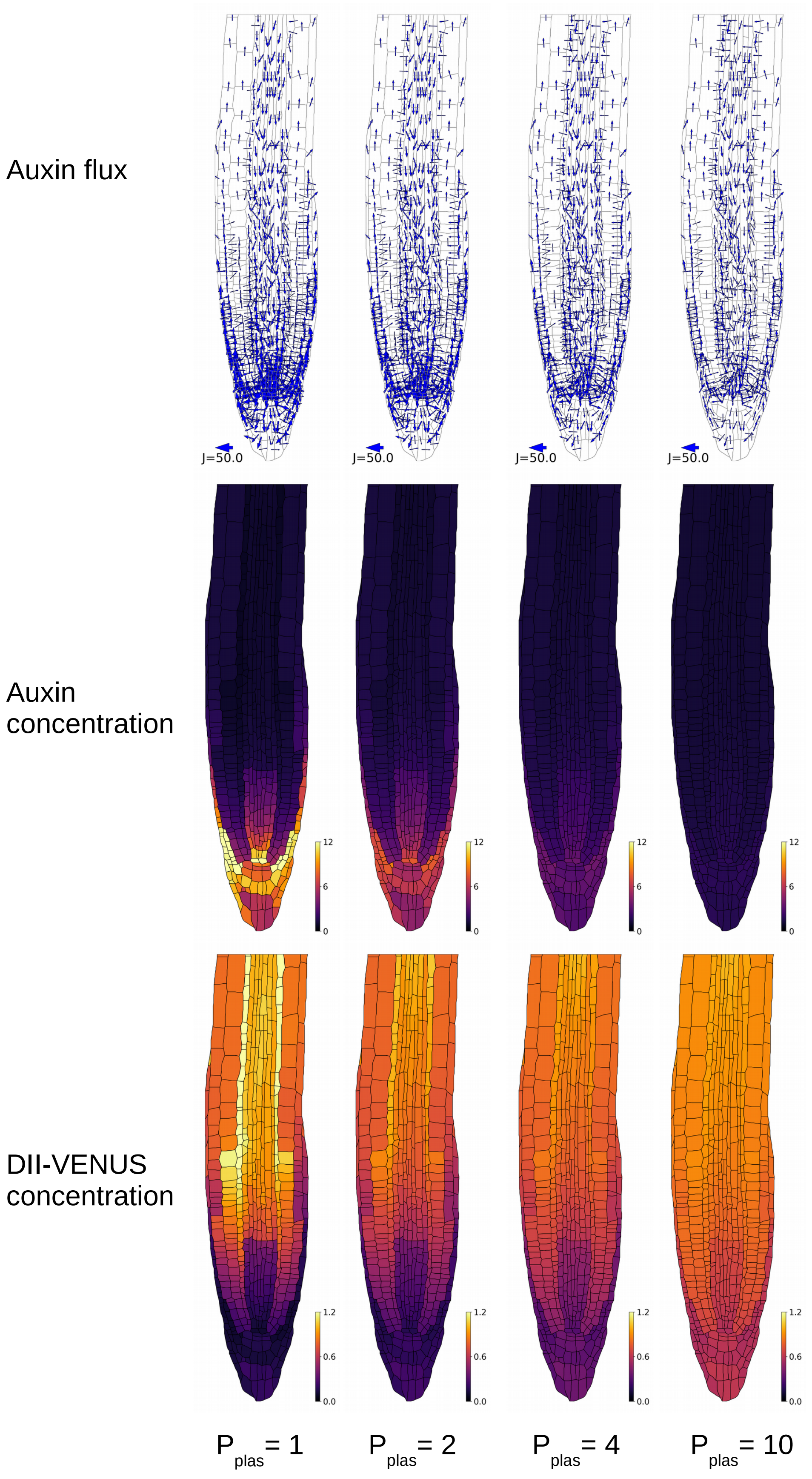

Figure S19: Effect of increasing plasmodesmatal permeability (P_{plas}) on predicted auxin flux (top), auxin concentration (middle) and DII-VENUS (bottom). The colour scales on each set of images are set the same for easier comparison between values of P_{plas}.

Development • Supplementary information

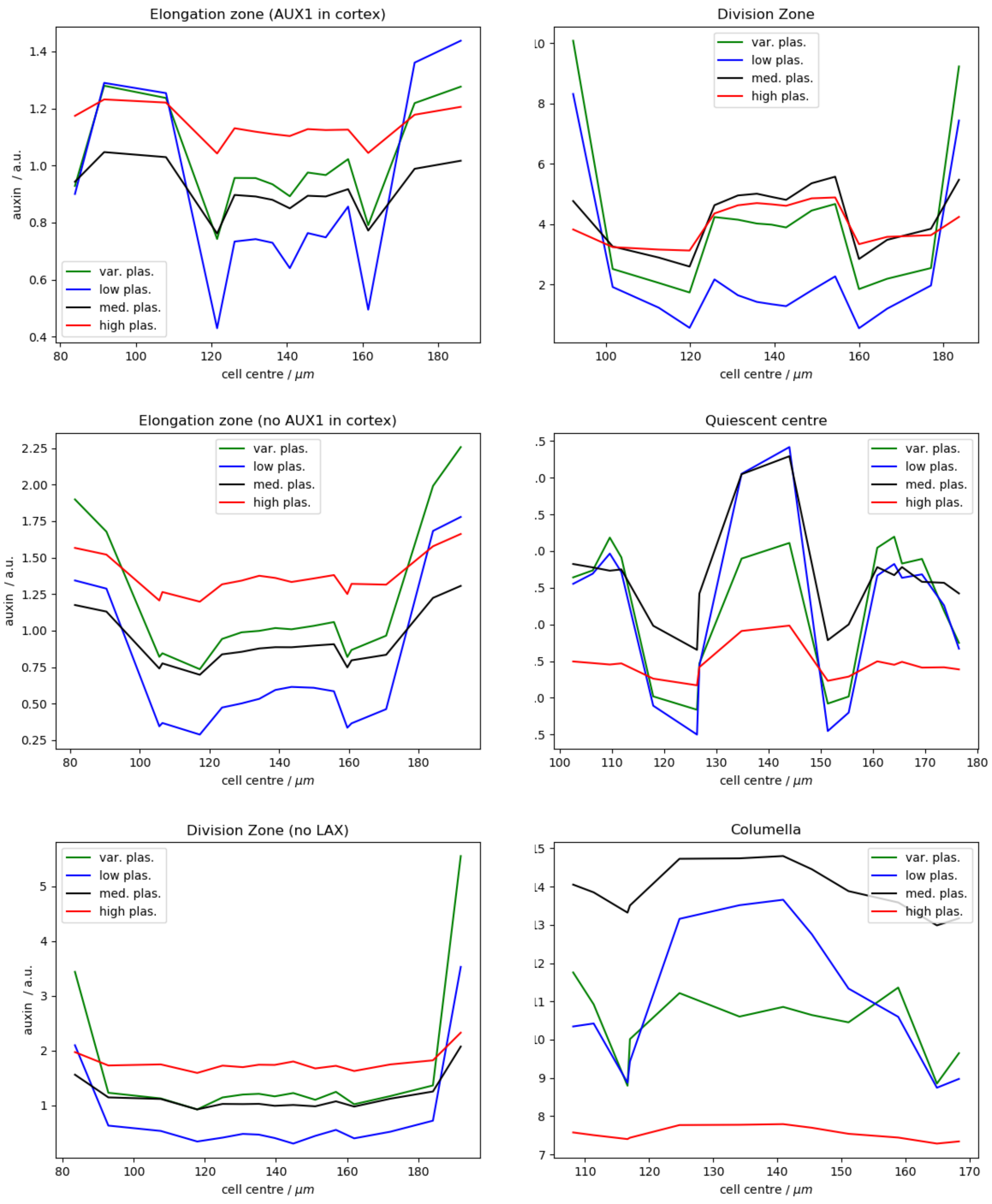

Figure S20: Horizontal cross sections of auxin concentration at various vertical positions in the root tissue for heterogenous plasmodesmata model (see Figure 2A for distribution), and models with uniformly low (0.83 μm^{-2}), medium (5.42 μm^{-2}) and high (12.58 μm^{-2}) plasmodesmatal densities.

Development • Supplementary information

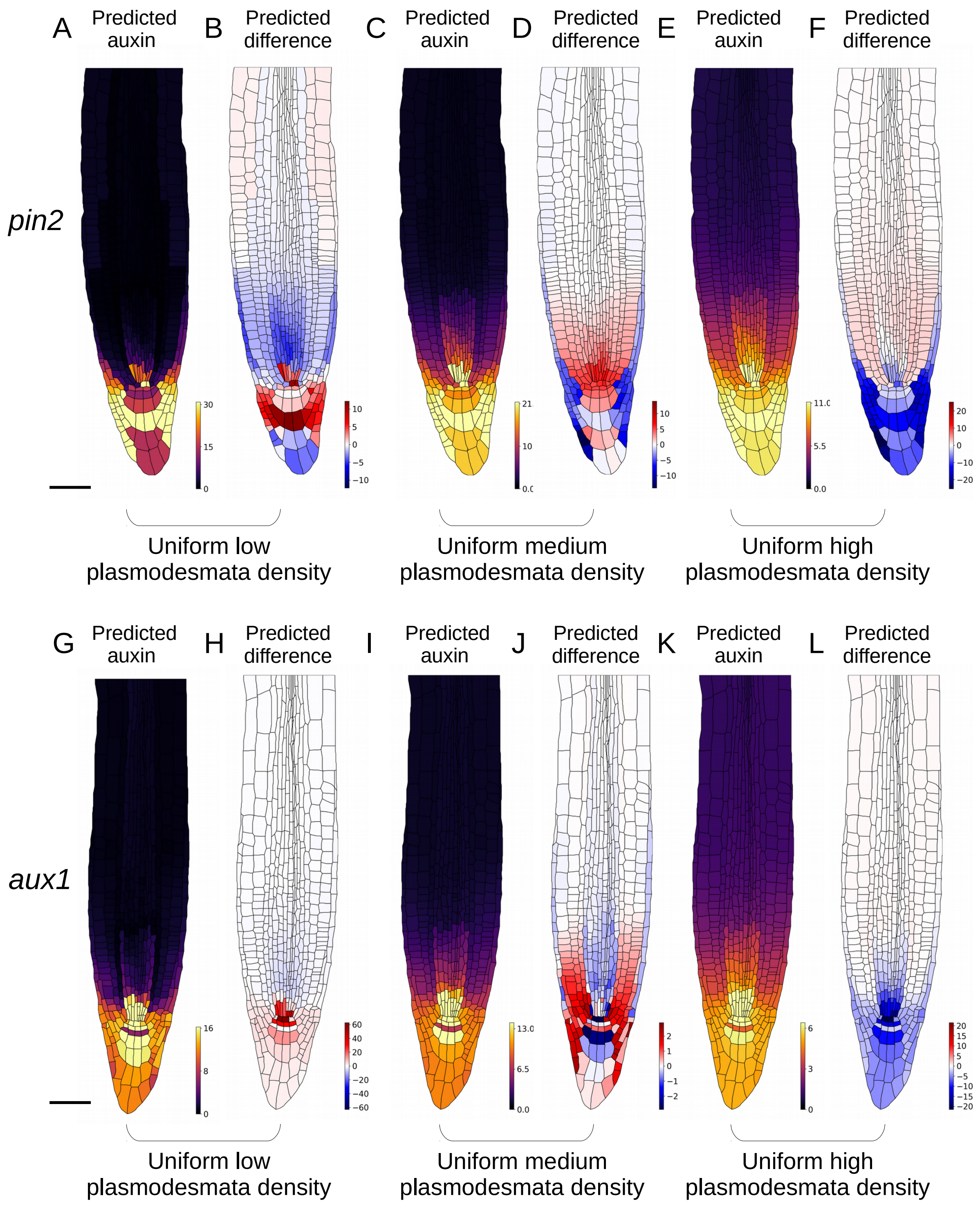

Figure S21: Root tip auxin distribution with uniform plasmodesmata density in *pin2* (A-F) and *aux1* (G-L). The predicted differences shown are between the auxin concentrations for the respective uniform plasmodesmata models and the variable plasmodesmata model (as shown in Figure 2F for *pin2* and Figure S4G for *aux1*). (A,B,G,H) Low plasmodesmatal density (0.83 μm^{-2} as in as in periclinal walls between lateral root cap and epidermis); (C,D,I,J) Medium plasmodesmatal density (5.42 μm^{-2} as in anticlinal epidermal walls); (E,F,K,L) High plasmodesmatal density (12.58 μm^{-2} as in anticlinal endodermal walls). Scale bar 50 μm.

Development • Supplementary information

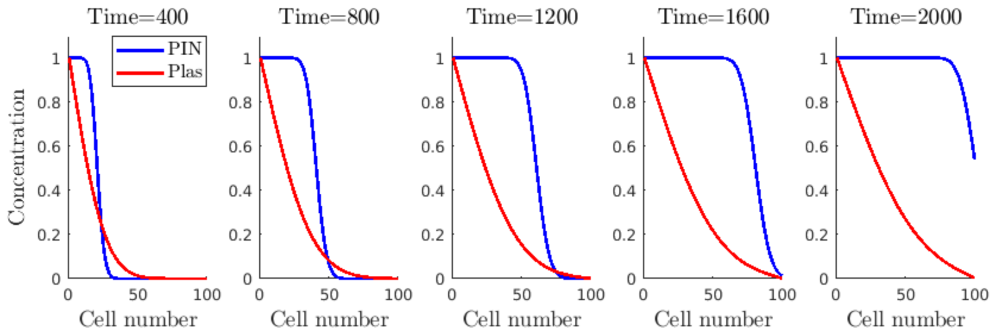

Figure S22: Effect of plasmodesmata on auxin propagation through a single file of cells. We suppose that auxin moves across cell membranes via both passive diffusion of protonated auxin and active transport mediated by PINs that, when present, are located polarly on the downstream membrane face of each cell. We suppose that auxin also passively diffuses between adjacent cell cytoplasms through plasmodesmata (in the case where plasmodesmata are present). See SI section 2.6 for the model equations. ($P_{plas} = 10\ \mu m^3\ s^{-1}$)

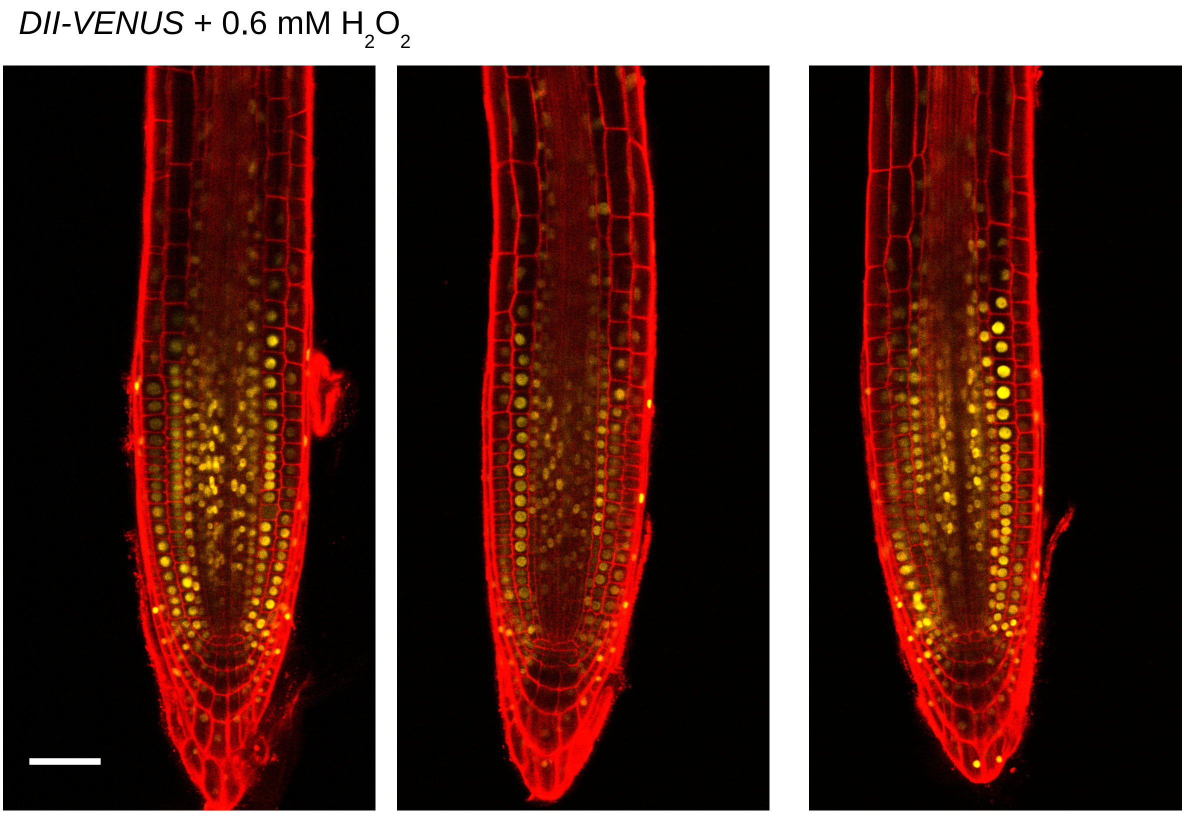

Figure S23: Replicates of background DII-VENUS distribution following 0.6 mM H_2O_2 treatment. Scale bar 50 μm.

Development • Supplementary information

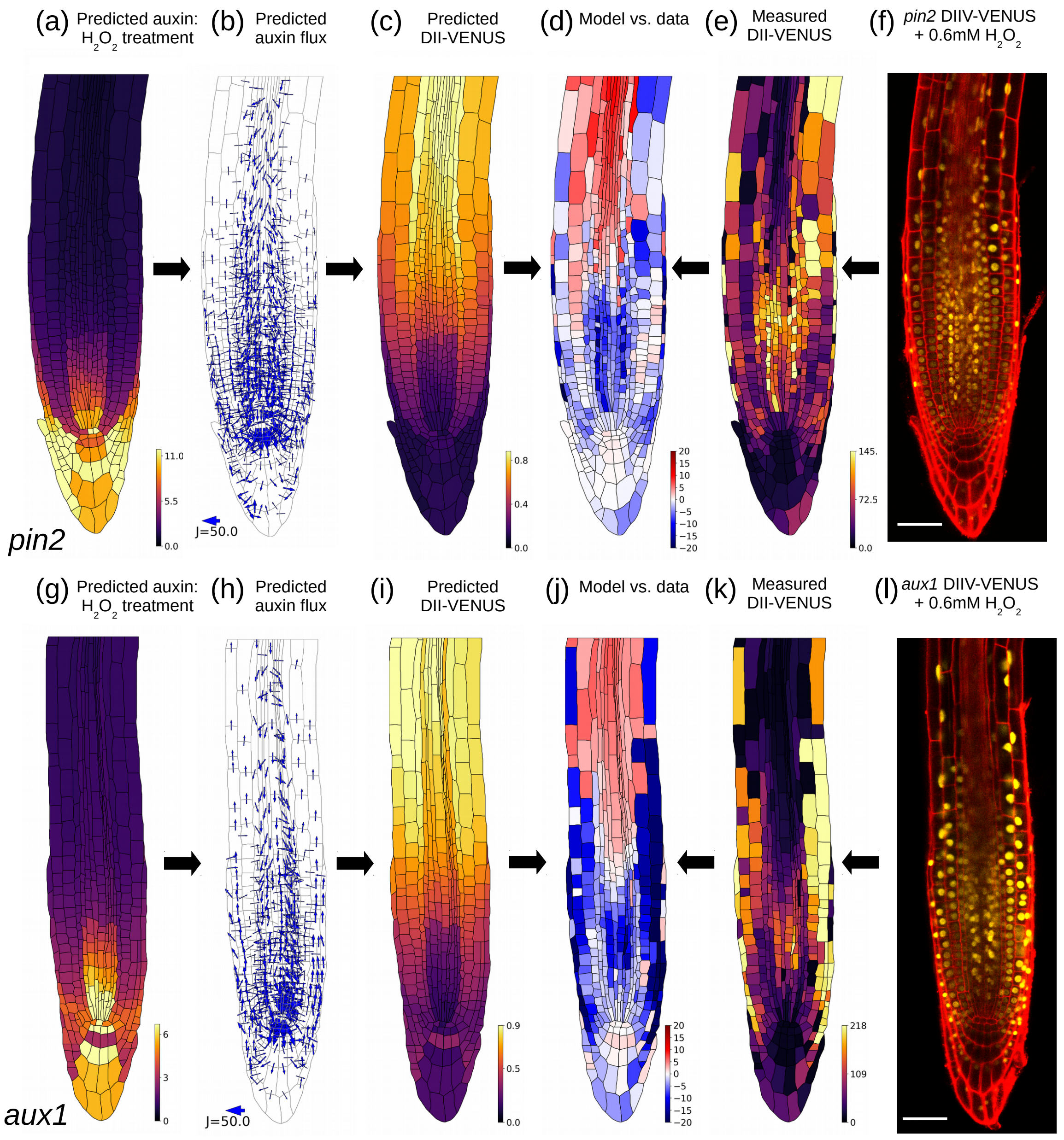

Figure S24: Effect of 0.6 mM H_2O_2 treatment on the distributions of *pin2* (a-f) and *aux1* (g-l) auxin and DII-VENUS. (a,g). Predicted steady-state auxin distribution (b,h). Predicted auxin fluxes (c,i). Predicted DII-VENUS distribution (d,j). Difference between predicted and observed DII-VENUS distribution (from predictions in panels c,i and data in panels e,k). (e,k). Quantification of DII-VENUS distribution using images in panels f and l. (quantified using CellSet image segmentation software). (f,l). Representative DII-VENUS confocal images. Scale bars 50 μm.

Development • Supplementary information

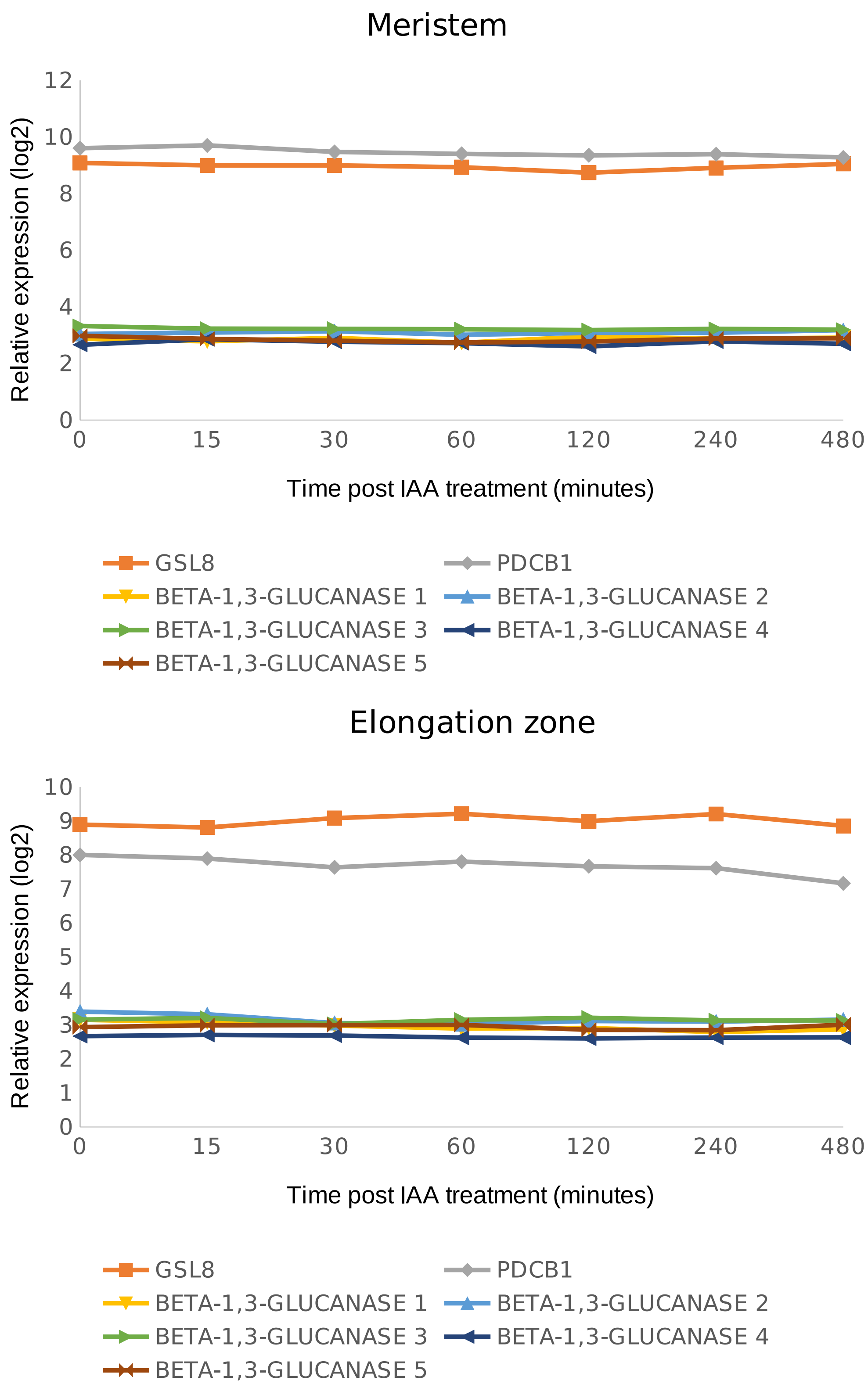

Figure S25: Time series transcriptomic analysis of key genes involved in callose regulation in the *Arabidopsis* root meristem (top) and elongation zone (bottom) post 1 μM IAA treatment (data reproduced from Voß *et al.* 2015, see this for details.)

Development • Supplementary information

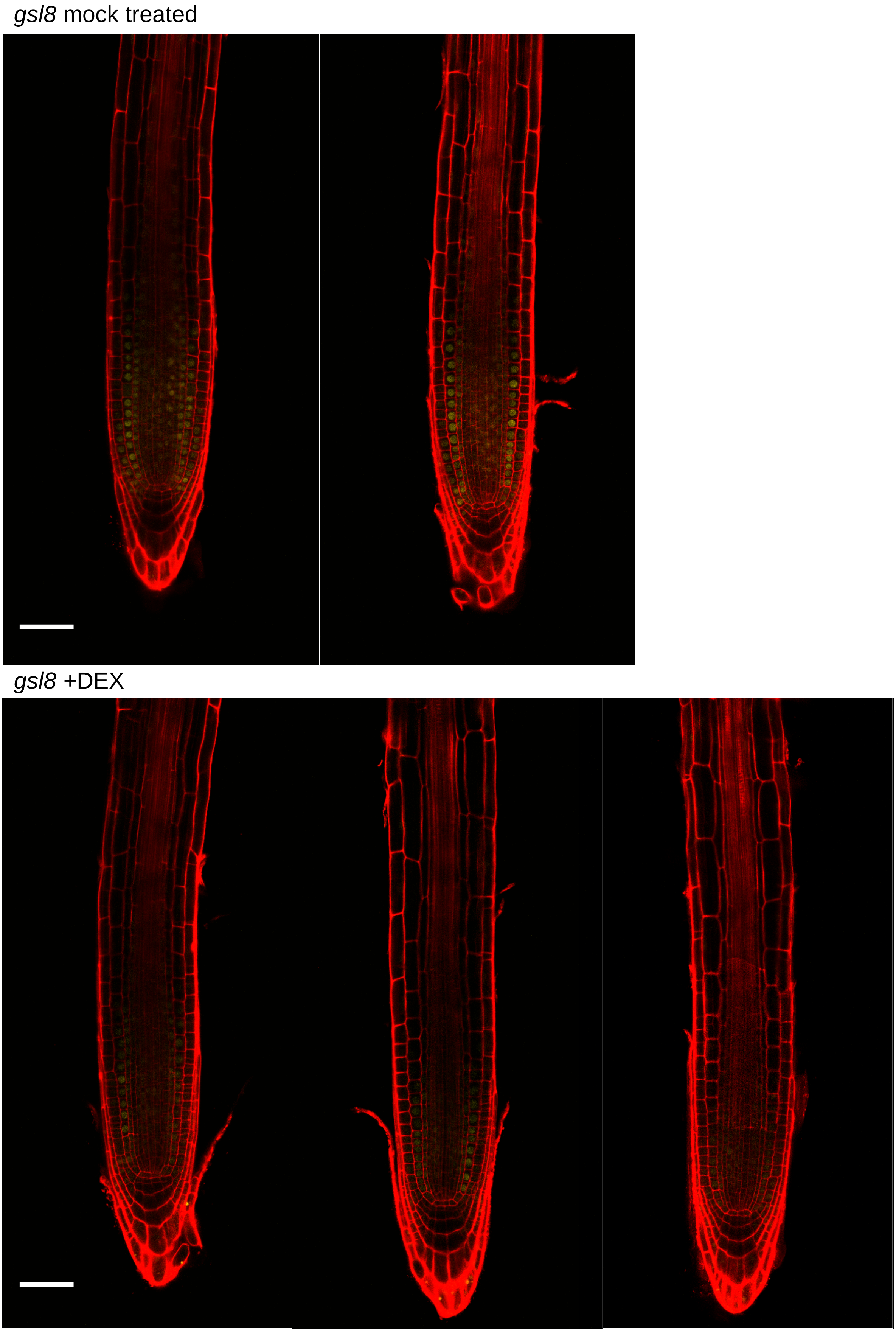

Figure S26: Replicates of mock treated *gsl8* DII-VENUS roots (top) and 25 hour DEX treated *gsl8* DII-VENUS roots (bottom). Scale bars 50 μm.

Development • Supplementary information
